# Black carbon structuring marine microbial activities and interactions: a micro- to macro-scale interrogation

**DOI:** 10.1007/s11356-025-36603-0

**Published:** 2025-06-13

**Authors:** Amira Saidi, Luca Zoccarato, Giovanni Birarda, Xavier Mari, Markus Weinbauer, Lisa Vaccari, Mauro Celussi, Francesca Malfatti

**Affiliations:** 1 Oceanography Section, National Institute of Oceanography and Applied Geophysics – OGS, Via Auguste Piccard, 54, Trieste, 34151 Italy; 2 Department of Environmental Sciences, Informatics and Statistics Ca’Foscari Università di Venezia, Venice, Italy; 3 Institute of Computational Biology, University of Natural Resources and Life Sciences (BOKU), Muthgasse 18, Vienna, 1190 Austria; 4 Core Facility Bioinformatics, University of Natural Resources and Life Sciences (BOKU), Muthgasse 18, Vienna, 1190 Austria; 5 Elettra – Sincrotrone Trieste, Strada Statale 14 - km 163,5 in AREA Science Park, Basovizza, Trieste, 34149 Italy; 6 Mediterranean Institute of Oceanography - MIO, Marine Environment Chemistry (CEM), Aix Marseille Université, Université de Toulon, CNRS, IRD, 163 avenue de Luminy - Bâtiment Méditerranée, Marseille, 13288 France; 7 Laboratoire d’Océanographie de Villefranche LOV, Institut de la Mer de Villefranche IMEV, Sorbonne Universités, UPMC, Université Paris 06, CNRS, 181 Chemin du Lazaret, Villefranche-Sur-Mer, 06230 France; 8 Life Sciences Department, Università Degli Studi Di Trieste, Via Fleming 22, Trieste, 34127 Italy

**Keywords:** Black carbon, Prokaryotes, Viruses, Enzymatic activities, Prokaryotic carbon production, 16S rRNA gene amplicon, FT-IR, AFM

## Abstract

Black carbon (BC) consists of partially combusted organic matter deriving from biomass and fuels burning. According to the IPCC’s reports, BC emissions are the second-largest contributor to global warming after CO_2_. BC enters the marine system via dry deposition or river run-off. Once in the sea, BC has the potential to affect nutrient biogeochemical cycles. In a series of four incubation experiments (Adriatic Sea and Ligurian Sea) and a pilot study, we have challenged the microbes with heavy loads of BC (24 mg L^−1^) in order to study the short-term BC effect on microbial dynamics and activities. Upon BC amendment, heterotrophic prokaryotes increased in abundance while viruses decreased. At the microscale, microbes became attached to BC particles, very heterogeneous in shape and size and enriched in proteins over time; these findings were confirmed by Fourier transform-IR spectroscopy and atomic force microscopy. Enzymatic degradative activities, proteases, and alkaline phosphatases were suppressed in the BC treatments despite an enhancement in prokaryotic carbon production. The 16S rRNA gene amplicon sequencing analysis did not show a significant shift in the microbial communities. Despite this, indicator species analysis revealed that *Arcobacter* and *Pseudoalteromonas* genera were statistically associated with the BC treatment at 48 h, thus suggesting their adaptive strategies to utilize BC. Our findings reveal that BC has the potential to stimulate intense carbon flow through microbial activity in the sea. Future studies should take account of the contribution of anthropogenic carbon, BC, into the marine biogeochemical C cycle.

**Supplementary Information:**

The online version contains supplementary material available at 10.1007/s11356-025-36603-0.

## Introduction

Black carbon (BC) is a general term that describes partially combusted organic matter (aerosol and particles) deriving from biomass and fossil fuels burning (Bond et al. 2013). Within the combustion continuum (Wagner et al. 2021), BC is hydrophobic and absorbs light, displaying diverse degrees of solubility in water. BC particles are structurally very heterogeneous in shape and size, and their composition is very complex, besides being enriched with aromatic compounds (Trilla-Prieto et al. 2021; Taylor et al. 2020; Liu et al. 2020; Lin et al. 2015; Zhang et al. 2013; Dittmar et al. 2008). According to the IPCC’s reports, BC emissions are the second-largest contributor to global warming after CO_2_ emissions (IPCC, 2014; IPCC, 2023) and lately BC global trend has been estimated to be decreasing as reported in the “Short-lived Climate Forcers” from the SIX Assessment Report IPCC (Szopa et al. 2021). Overall, BC is a very pervasive force shaping the atmosphere dynamics, climate (Bond et al. 2013; Haywood and Shine 1995), and represents a major issue for human health (e.g., respiratory diseases; Bond et al. 2013; Haywood and Shine 1995; IPCC, 2014; IPCC 2023).

BC is produced mostly inland and its flux into the marine environment is about 29 Tg C per year (Wagner et al. 2018; Jurado et al. 2008; Bao et al. 2017), thus influencing the carbon biogeochemistry in the ocean (Coppola et al. 2022). It has been estimated that BC particles enter the marine system via dry deposition (BC load, 1.8 ± 0.83 Tg year^−1^; Wu et al. 2018; Jurado et al. 2008; Bao et al. 2017; Katz et al. 2024) or river runoff (BC load, 26 Tg year^−1^; Jaffé et al. 2013; Mari et al. 2017). The small particles of BC are dissolved in the ocean (DBC) and join the dissolved organic carbon (DOC) pool before reaching the sediment (Masiello and Druffel 1998). The BC burial magnitude contributes for 1.5–3.3% of the ocean’s anthropogenic CO_2_ uptake (Yamashita et al. 2022). Based on isotopic analysis, it has been found that δ13 C is different between riverine and oceanic DBC (Wagner et al. 2019). This has led to the hypothesis that other sources could contribute to the DBC pool, such as the marine phytoplankton. Furthermore, another hypothesis proposes that DBC is transformed by bacteria, enters the C cycle, and ages during transport on similar timescales as DOC in the ocean (Qi et al. 2020). Recently, a study showed that BC is biodegradable, and the diverse sources of BC play key roles in regulating microbial activity and consumption (Martinot et al. 2023). According to the source, BC chemical composition can boost microbial growth and fuel the C cycle (Martinot et al. 2023). BC exhibits different dynamics once it enters the marine environment. Studies in the Pacific Ocean (Yamashita et al. 2022) showed that DBC concentrations decrease with depth, thus highlighting a possible transfer of DBC from the deep ocean to the abyssal sediments via sinking particle adsorption. Overall, the deep ocean can then be considered as a sink for BC (Suman et al. 1997).

Once they enter the lit ocean, BC particles have the potential to structure the organic matter continuum and the microbial-mediated carbon biogeochemical cycle (Weinbauer et al. 2017). Notably, BC enriches productive surface waters with carbon and leads to aggregation processes, resulting in shifts in nutrient concentrations and dynamics (Mari et al. 2014, 2019). Given their chemical composition, BC particles adsorb organic matter, such as amino acids, that subsequently promote microbial colonization (Benavides et al. 2019). Once these particles are colonized by microbes, they may be remineralized as an integral part of the C biogeochemical cycle. BC particle deposition can also decrease the pH of seawater, thus, representing a source of ocean acidification without affecting the pCO_2_ (Weinbauer et al. 2012). Under changing climate, with more frequent severe wildfires and sustained fossil fuel emission of BC particles in the atmosphere, BC effect on the marine microbial ecosystem and global carbon biogeochemical cycles is thought to surge. We now know that BC acts as an environmental sieve for microbes and potentially affects other marine organisms (Ventura et al. 2021).

Microbes are abundant in the ocean (~ 10^8–9^ cells/liter, Munn, 2011), very diverse (Sunagawa et al. 2015), and a major driver of organic matter degradation (Azam and Malfatti 2007; Banerjee et al. 2018). At the microscale, bacteria interact with all living and nonliving particles present in the sea, thus including BC (Azam and Malfatti 2007; Mari et al. 2014; Cattaneo et al. 2010). Previous studies have shown that in the sea, BC particles act as a sponge absorbing marine viruses, bacteria, and organic matter (Cattaneo et al. 2010; Mari et al. 2014, 2019). BC has also been demonstrated to stimulate bacterial production (Mari et al. 2014; Martinot et al. 2023). Furthermore, BC has been shown to promote high viral lytic infection rates, especially in the sea surface microlayer, that is, the uppermost atmosphere–ocean interface (Ram et al. 2018; Cunliffe et al. 2013). Despite a lot is known about BC in the marine ecosystem, we still ignore mechanistic details on how microbial metabolisms affect the bioavailability of BC in the dissolved and particulate form along the combustion continuum (Wagner et al. 2021). Within the biogeochemical carbon cycle, a current knowledge gap is the lack of data on microbial degradative activities on marine organic matter pools.

The aim of this paper was to deepen the knowledge of marine microbe-BC interactions at the micro- and macro-scale within the biogeochemical carbon cycle. We focused on the dynamics of organic matter processing, microbial dynamics (i.e., extracellular enzymatic activities and prokaryotic carbon production), and microbial community composition upon BC challenging over time. Our overarching hypothesis was that BC boosts the biogeochemical carbon cycle by influencing microbial dynamics. This implies that (1) the organic matter degradation and prokaryotic C production were enhanced in BC; (2) the microbial community diversity and structure were affected by BC deposition thus promoting the emergence of BC-specific taxa; and (3) BC particles are very diverse in structure and change chemically over time.

To test these hypotheses, we set up controlled BC-enriched seawater culture incubation experiments (Hagström et al. 1984; Ammerman et al. 1984; Weinbauer et al. 2012) in two coastal areas in the Mediterranean Sea that are subjected to intense and variable BC depositions due to the wind regimes and the industrial point source activities in the vicinity (European Environmental Agency portal and Copernicus CAMS for PM 10 and PM 2.5).

## Materials and methods

### Study areas

In this study, we have explored microbial dynamics, activities, and community structure in two contrasting Mediterranean coastal areas: the Adriatic and the Ligurian Sea (Table S1), which exhibit distinct trophic status. The North Adriatic Sea biogeochemical dynamics are heavily influenced by seasonal changes in the water column thermal stability, highly variable freshwater inputs, and wind-driven sediment resuspension (Celussi and Del Negro 2012; Manna et al. 2021). Chlorophyll *a* concentrations generally range between 0.5 and 1.1 μg L^−1^, and microbial dynamics are influenced by temperature and substrate colimitation (Manna et al. 2021). Sedimentation fluxes of organic carbon are high, up to 4.6 kg C m^−1^ year^−2^ (Giani et al. 2018 and ref therein). In contrast, the Ligurian Sea is characterized by lower nutrient concentrations, low chlorophyll, and by little or no river discharges (Migon 1993). The microbial dynamic is structured by bottom-up and top-down controls, mainly phage lysis and dissolved organic matter (DOM) input (Tanaka and Rassoulzadegan 2004). Recent data on the chlorophyll trend in the Ligurian Sea showed a decrease starting after 2000 (Vandromme et al. 2011). In comparison to the Adriatic sedimentation rate, the Ligurian sedimentation rate of organic carbon accounts for a grand total of 21 g cm^−1^ year^−2^ (Miquel et al. 1994; 2011). Within these diverging microbial ecosystems, we have investigated the BC effect on microbial dynamics, functions, and community structure.

### Sampling design

Twenty liters of raw seawater were collected either by means of Niskin bottles or with an acid-clean bucket from four locations: one coastal and one open-water station both in the northern Adriatic Sea and in the Ligurian Sea (Table S1). In each experiment, the water was pre-filtered onto 3-µm sterile polycarbonate filter membrane to remove phytoplankton cells, and the filtrate was distributed in 6 acid-clean, 2-L polycarbonate bottles. Three bottles were maintained as control (CTRL), while the remaining three bottles were amended with black carbon to a final concentration of 24 mg L^−1^ (BC). Our BC concentration is within the range of other studies: Malits et al. (2015) used 20 and 100 mg L^−1^, Cattaneo et al. (2010) used 10 mg L^−1^, Ram et al. (2018) used 20 mg L^−1^, Mari et al. (2014) used 0.2–0.9 mg L^−1^, and Martinot et al. (2023) used 21. 27 and 47 mg L^−1^.

The black carbon powder used in this study was black carbon standard material lignocellulosic char: risotto rice (origin Switzerland, from Department of Geography, Soil Science and Biogeochemistry, Universität Zürich, Switzerland). As reported in Hammes et al. (2007), the BC risotto rice was derived from the combustion of *Oryza sativa* at 450 °C.

for 5 h, in a nitrogen atmosphere. The organic carbon content was 591.4 g kg^−1^mass, and its surface area was equal to 5.9 m^2^ g^−1^. BC powder was insoluble, and it was homogeneously mixed in the bottle. During all the incubation experiments, all the bottles were incubated constantly in the dark at in situ temperature (22 °C) in a constant motion within the water incubator. Over the course of the experiments, the water was sampled for microbial abundances, extracellular enzymatic activities, and prokaryotic carbon production (only in experiment 1, Adriatic Sea). Samples were taken from each bottle at time zero (T0) and after 2, 6, 24, and 48 h. For the BC treatments, the T0 was considered after 30 min from the BC amendment. BC bottles were gently inverted 3 times, in order to allow the powder to mix within the seawater. At the beginning and at the end, we sampled water for the 16S rRNA gene amplicon sequencing analysis for microbial community diversity and structure. We choose to run all the incubation in the dark in order to focus on organic matter degradation and prokaryotic carbon production without being biased by the newly primary production. Exploratory microscale interrogations were performed by AFM during a pilot study (SI) and within the Adriatic Sea experiment by FT-IR (SI). Clinical trial number: not applicable for this study.

### Prokaryotic and viral abundance

The quantification of heterotrophic prokaryotes (HP), *Synechococcus* (SYN), and viruses (as viral-like-particles—V) was investigated using flow cytometry in accordance with the methods established by Gasol et al. (1999) and Brussaard (2004). The instrument utilized was a FACSCanto II (Becton Dickinson), equipped with a standard filter setup and a 488 nm air-cooled laser. Samples (1 mL) were fixed with 0.5% glutaraldehyde solution final concentration (Grade I for EM analyses, Sigma Aldrich). Fixed samples were kept at 4 °C for approximately 20 min and then stored at − 80 °C until analysis. Prior enumeration, samples were thawed at room temperature and diluted 1:10 (HP) and 1:50 (V) with 0.2 μm-filtered 1X Tris–EDTA buffer. Then, samples were stained with SYBR ™ Green I nucleic acid dye, following the staining procedures outlined by Marie et al. (1999) and Brussaard (2004) for HP and V, respectively. HB were stained (1 ×) and incubated for 10 min in the dark at room temperature. V were stained (0.5 ×) and incubated for 15 min in the dark at 80 °C. The counts of SYN were measured using undiluted, unstained seawater samples. Heterotrophic prokaryotes and viruses were identified based on their green fluorescence and side scatter with a threshold on the 488-nm laser. *Synechococcus* cells were identified based on their autofluorescence in the chlorophyll *a* and phycoerythrin channels. The abundance of BC-attached prokaryotes was established, according to Gasol and Moran (2015) using the same HB settings in terms of voltage and threshold. In the side scatter vs green fluorescence cytogram, BC-attached prokaryotes appeared as a population of events with high green fluorescence and large side scatter values. Blanks were run with unstained BC powder to confirm the position of the BC particles in the cytogram along the side scatter axes. Abundances were calculated using the acquired cell counts and the respective flow rates. Given the experimental settings (i.e., dark incubation), we were not able to detect any BC effect on the unicellular primary producers: *Synechococcus* (Table S2, Table S3, Fig. S1).

### Extracellular enzymatic activities

The assessment of extracellular enzymatic activities was carried out in line with the method of fluorogenic substrate analogues, as detailed by Hoppe (1993). The fluorophores employed in this procedure were 7-amino-4-methyl-coumarin (AMC) and 4-methyl-umbelliferone (MUF). The hydrolysis rate of leucine-AMC and MUF-phosphate were used to assay leucine aminopeptidase and alkaline phosphatase, respectively. The activities of these enzymes were then expressed in terms of the MUF or AMC production rate over time. Saturating concentrations were established by testing varying substrate concentration while measuring rate of hydrolysis to find the maximum speed (Celussi and Del Negro 2012; Celussi et al. 2017; Van Wambeke et al. 2021). Hydrolysis rate was measured by incubating for 1 h in the dark at in situ temperature: the 2.5-mL sub-samples with 200 µM, leucine-AMC for both basins, and 50 µM MUF-phosphate in the Adriatic locations and 25 µM MUF-phosphate in the Ligurian locations. The hydrolysis of AMC and MUF from the model substrates was identified by increased fluorescence levels, which were measured with a Shimadzu RF-1501 spectrofluorometer (AMC = 380 nm excitation and 440 nm emission; MUF = 365 nm excitation and 455 nm emission). Triplicate blanks without fluorogenic substrate were utilized to determine the natural fluorescence increase in the samples unrelated to the enzymatic activity. Calibration curves were constructed using 0.2 µm-filtered seawater, with standard solutions of MUF and AMC (5 µM each) added in triplicate (Table S2).

### Prokaryotic carbon production

The determination of prokaryotic carbon production was conducted via the incorporation of ^3^H-leucine (Leu), following Kirchman et al. (1985). Both triplicate aliquots, each containing 1.7 mL, and two controls obtained with the addition of 90 µL of 100% trichloroacetic acid (TCA) were amended with 20 nM radiotracer and incubated at in situ temperature under dark conditions. After 1 h, the incubation process was stopped by introducing TCA (at a final concentration of 5%). The extraction process was done using 5% TCA and 80% ethanol, utilizing the microcentrifugation technique as detailed by Smith and Azam (1992). Sample activity was investigated using a β-counter (TRI-CARB 2900 TR Liquid Scintillation Analyzer) following the addition of 1 mL of scintillation cocktail (Ultima GoldTM MV; Packard). Carbon biomass production was estimated using a conversion factor of 3.1 kgC mol^−1^ Leu (Table S2), based on the assumption of a twofold isotope dilution, as described by Simon and Azam (1989).

### Prokaryotic DNA extraction, sequencing, and bioinformatic analysis

Approximately 2L of water at T0 and T48 were filtered onto 0.2 µm PES filter membrane (Supor ®, 47 mm diameter PALL) and immediately stored at − 80 °C until further processing. DNA was extracted using the MO BIO’s PowerSoil DNA kit according to the manufacturer’s protocol and sent to the sequencing service LGC Genomics GmbH (Germany). The V4 region of the 16S rRNA gene was amplified using the primers 515 F (5′-GTGCCAGCMGCCGCGGTAA-3′) and 806R (5′-GGACTACHVGGGTWTCTAAT-3′) according to the Earth Microbiome Project protocols (Caporaso et al. 2012; Parada et al. 2016).

Bioinformatic analyses were performed with QIIME 2 2023.5 (Bolyen et al. 2019) on demultiplexed reads received from the sequencing service company. Adaptor sequences and primers were removed from the paired-end reads using Cutadapt (Martin 2011) integrated within QIIME 2. Based on the visual inspection of quality profiles, forward and reverse raw sequences were then trimmed at 240 bp and 140 bp, respectively. Reads were then filtered applying the default values and denoised using the DADA2 (i.e., method, independent; Callahan et al. 2016). The taxonomy was assigned to each amplicon sequence variant (ASVs) using the sklearn QIIME2 naive Bayes taxonomy classifier (Bokulich et al. 2018) against the Silva 138.1 NR99 (non-redundant at 99% identity) reference database with 7-level taxonomy (Quast et al. 2013). Reads belonging to Eukarya, mitochondria, chloroplasts, and ASVs with < 2 total counts (singletons) were removed.

Microbial diversity was explored and visualized in R (version 4.3.2) with the packages phyloseq 3.16 (McMurdie and Holmes 2012) and ggplot2 3.4.4 (Wickham 2016).

Rarefaction curves were obtained using the ggrare function within the vegan package (V 2.6.4). Alpha-diversity metrics were estimated using Shannon and Pielou indexes. An analysis of variance (ANOVA) was performed to test the significance between environmental and biological variables, followed by a normality check with QQplot to ensure that the residuals were normally distributed, an assumption for the validity of the ANOVA. The similarity patterns were visualized using a principal coordinate analysis (PCoA) based on Bray–Curtis dissimilarity matrices within R package vegan (Oksanen et al. 2022). The significance of dissimilarities was tested using Permanova (using adonis2 function), thus assessing the difference between the microbial communities across different locations (AO, AC, LO, LC, Table S1), with different treatments (BC, CTRL) at different incubation times. After converting the phyloseq object to relative abundances (RAs), taxa were agglomerated to genus level to visualize the most 20 abundant genera. We manually curated the four non-classified genera found among the most abundant 20 taxa by blasting them against the NCBI database. The indicator species analysis was performed with the R package indicspecies (V1.7.14) (De Cáceres and Legendre 2009). The indicator species analysis aims to identify the taxa that were significantly associated with specific groups of variables such as here treatment over time considering the indicator value that has *p*-value < 0.05. To calculate the index species, we used the function multipatt from the indicspecies package (9999 permutations and alpha = 0.05), utilizing the r.g index (Tichý and Chytrý 2006). An abundance-based analysis was performed, using the ASV counts to assess the strength and nature of associations.

### High-resolution analysis by FT-IR and AFM of BC

FT-IR: The BC samples collected from the Adriatic Open site incubation at the end of the experiment were measured at the infrared beamline of Elettra Sincrotrone Trieste, SISSi-Bio (https://doi.org/10.1117/12.2607751), using two different sampling methodologies: micro-attenuated total reflection and transmission in a diamond anvil cell (Diamond EX-Press, S.T. Japan Europe GmbH). Both acquisition modalities were carried out using a VIS-IR microscope Bruker Hyperion 3000, coupled with a VERTEX 70 V interferometer. Data were collected using either a single-point mercury cadmium telluride (MCT) detector and a 64 × 64 pixels focal plane array (FPA) imaging detector. The sampled area with the ATR setup is ~ 100 microns (Fig. S6), whereas in transmission the field of view of the detector is ~ 150 × 150 microns (Fig. S7).

AFM: Atomic force microscopy imaging was performed on a Dimension FastScan AFM (Bruker, Santa Barbara, CA USA) in PeakForce TappingTM Mode using ScanAsyst-Air probes (nominal spring constant 0.4 N/m and tip radius 5 nm, Bruker) for fixed and air-dried samples at the Scripps Institution of Oceanography, UCSD, in the Azam laboratory. The sample that has been imaged was from a BC spiked 3-µm-filtered seawater incubation after 24 h (see SI, Pilot experiment). The samples were prepared following Malfatti et al. (2009) in brief, 100 μL of formalin fixed sample from BC bottle was spotted onto cleaved mica, then let it dry and then washed with HPLC water. BC stock solution was also processed in the same way and imaged. Five scans per sample kind were acquired. Raw AFM height image data were processed using the nanoscope analysis built-in package (Bruker), with minimal line-flattening and plane-fitting functions.

### Statistical analysis

The treatment effect (CTRL and BC) on the biological variables heterotrophic prokaryotic abundance (HP), *Synechococcus* abundance (SYN), viruses abundance (VIRUSES), virus to bacteria ratio (VBR), enzymatic activities (Leu-AMA and APase activity), and prokaryotic carbon production (PCP) were assessed after normalization using paired Wilcoxon test (Hollander et al. 2015).

## Results

### Prokaryotic and viral dynamics

**Fig. 1:**
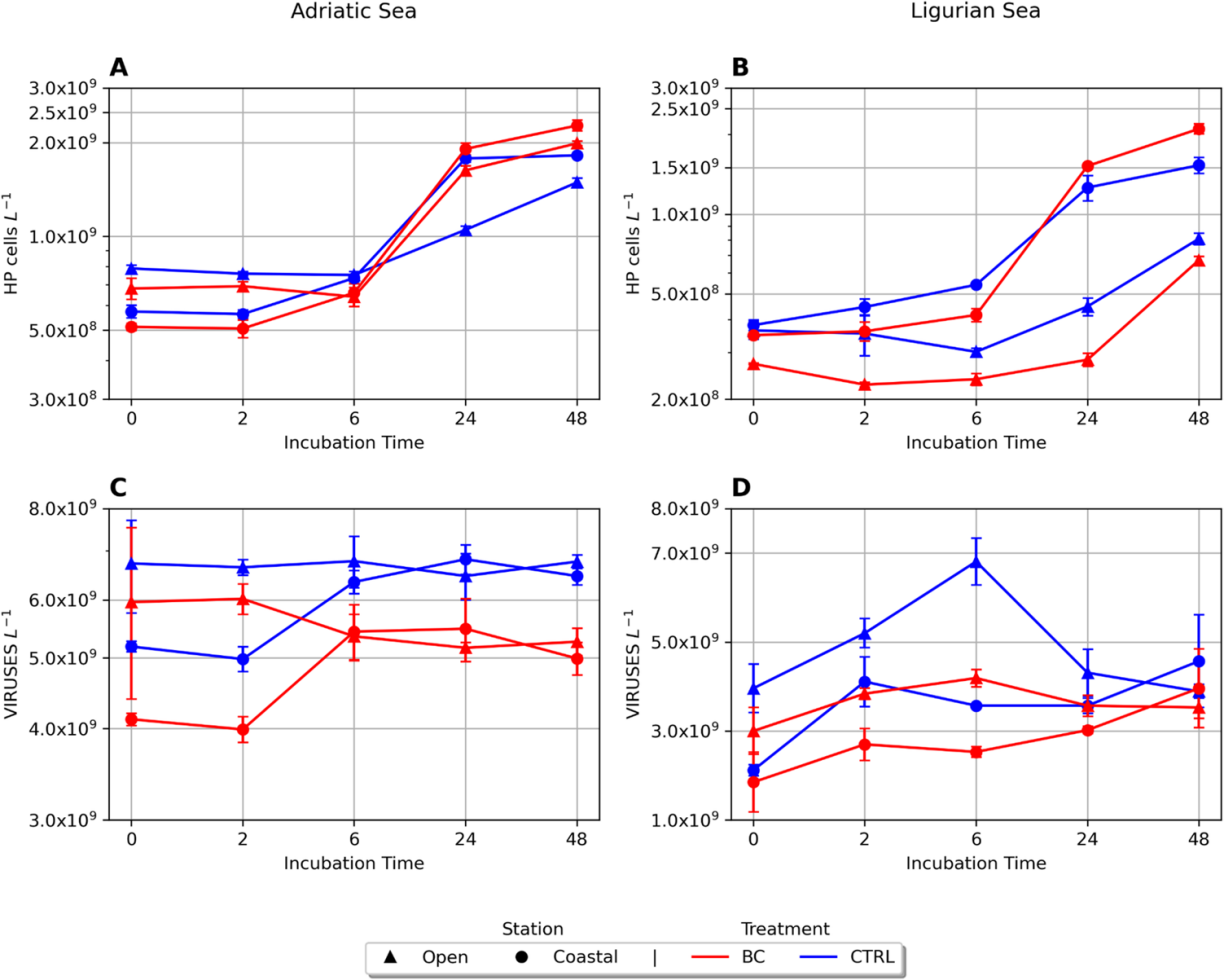
Heterotrophic prokaryote abundance (HP cells L^−1 ^; panels **A** and **B**) and viral abundance (viruses L^−1^; panels **C** and **D**) over incubation time in the different treatments. Color indicates treatment: black carbon (BC, red) and control (CTRL, blue). Shapes indicate stations: coastal (circle) and the open sea (triangle). Adriatic sea, left panel (**A** and **C**); Ligurian sea, right panel (**B** and **D**)

Microbial dynamics presented similar trends in all the experiments (Fig. 1). Heterotrophic prokaryotes increased in number over time in all enclosures, whereas no common trend was observed for viruses. Overall, when compared to controls, BC positively affected heterotrophic prokaryotes only in the Adriatic Sea incubations and in the Ligurian Sea coastal station, whereas BC negatively affected the viral population in both basins.

In the Adriatic experiments, at T0, heterotrophic prokaryote (HP) abundance ranged from 0.51 to 0.68 10^9^cells L^−1^ (Fig. 1). Over time, in all the treatments, prokaryotic abundance rose about 2.5 times.

**Fig. 2:**
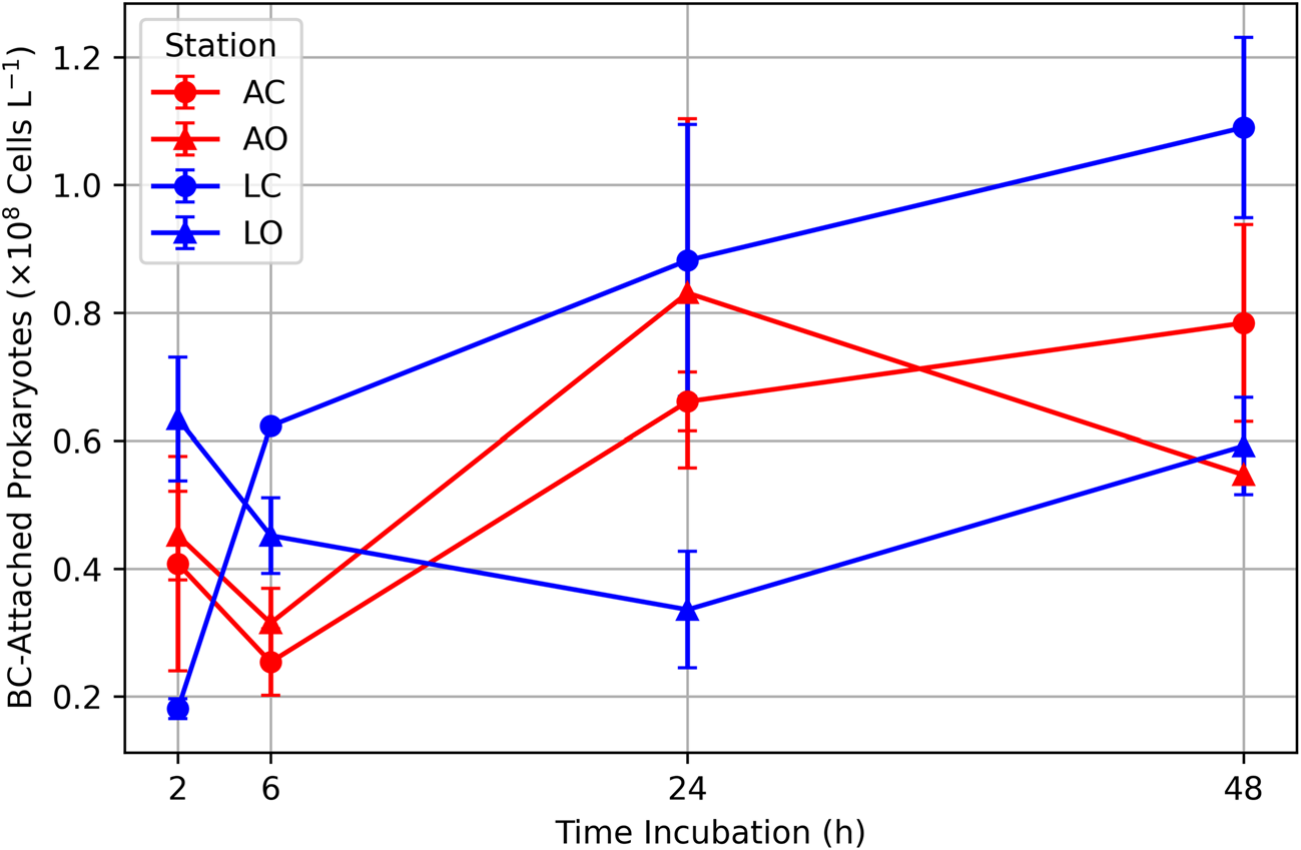
BC-attached heterotrophic prokaryotes over time in the Adriatic (red: AO, open sea; AC, coastal) and Ligurian Sea (blue: LO, open sea; LC, coastal) incubations

In the Ligurian experiments, at T0, heterotrophic prokaryote (HP) abundance ranged from 0.27 to 0.38 10^9^ cells L^−1^ (Fig. 1). After 24 h, heterotrophic prokaryotes and viruses (see below) were promptly attached to BC particles in all experiments (Fig. 2) since we collected after 30 min of BC amendments. Overall, attached prokaryotes increased over time in 3 out of 4 incubations, displaying highly dynamic trends. Also, *Synechococcus* became attached to BC particles (Table S2, Fig. S2).

In all experiments, the abundance of *Synechococcus* cells remained stable within 24 h then decreased at 48 h possibly due to the fact that the incubations were run in the dark (Table S2, Fig. S1). Overall, SYN abundances in the Adriatic Sea at T0 ranged from 4.33 to 5.92 10^7^ cells L^−1^ and in the Ligurian Sea at T0 from 1.08 to 1.36 10^7^ cells L^−1^.

Consistently, we found that viral abundance in BC was lower than in the CTRL treatments (Fig. 1, Table S2, Table S4). These differences were statistically significant in both the Adriatic and Ligurian basins (paired test Wilcoxon, V-statistic = 0, *p* < 0.001). Virus to bacteria ratio (VBR) presented similar trends in all treatments of AO, AC, and LC. VBR ranged between 5 and 9 in the first day and then it consistently decreased. In LO, VBR greatly increased within 6 h (BC-max = 17.60; CTRL-max = 22.55) and then abruptly decreased within 2 days (Fig. S1).

### Extracellular enzymatic activities and prokaryotic carbon production

**Fig. 3:**
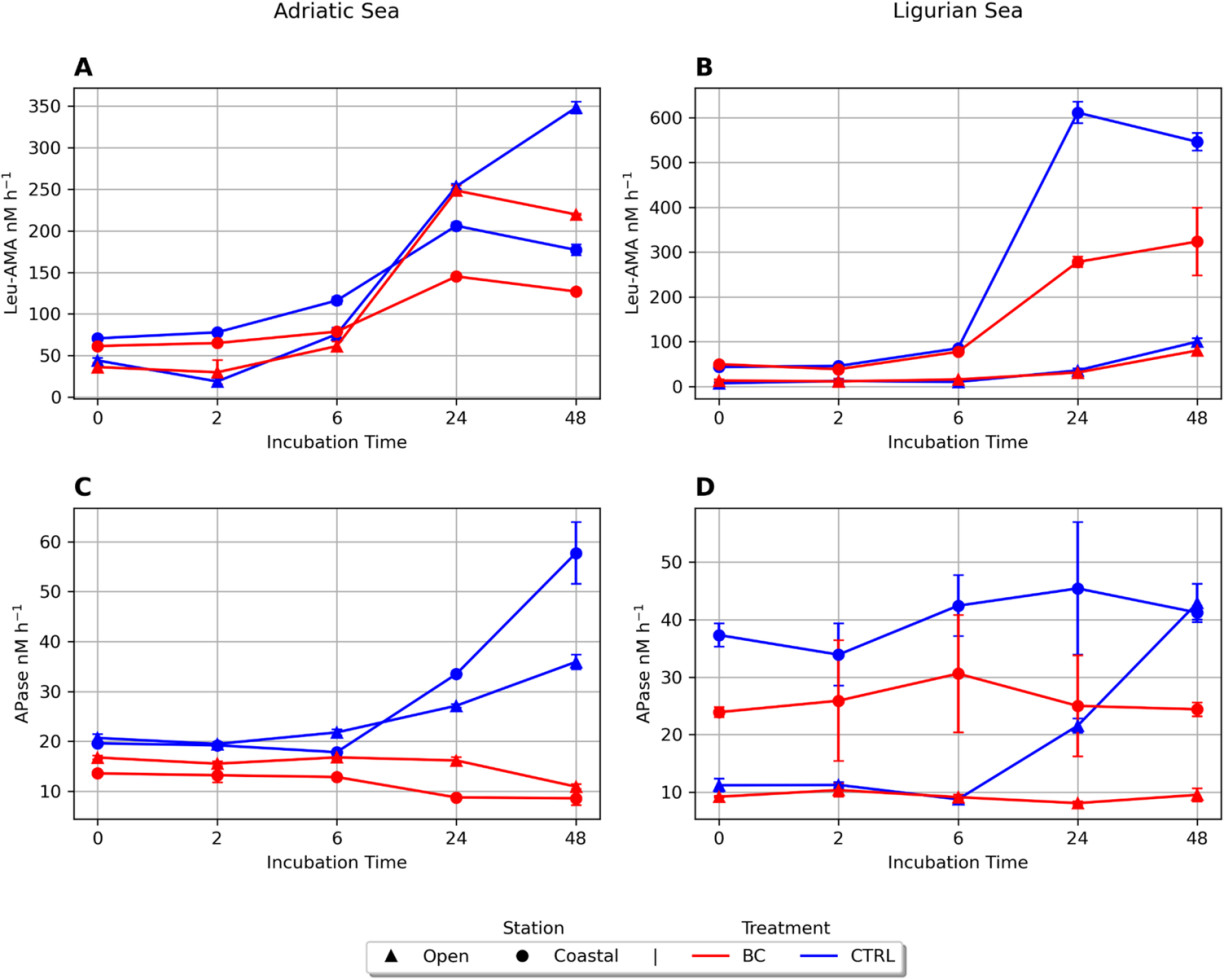
Extracellular enzymatic activities: leucine aminopeptidase (Leu-AMA, panels **A** and **B**) and alkaline phosphatase activity (APase, panels **C** and **D**). Units are expressed as nMh^−1^. Color indicates treatment: black carbon (BC, red) and control (CTRL, blue). Shapes indicate stations: coastal (circle) and the open sea (triangle). Adriatic sea, left panel (**A** and **C**); Ligurian sea, right panel (**B** and **D**)

In the Adriatic Sea, leucine aminopeptidase activity at T0 ranged from 36.32 to 70.82 nMh^−1^, remained stable for 6 h, and then sharply increased after 24 h in all treatments and only for the control AO the activity kept increasing at 48 h (Fig. 3). BC treatments inhibited leucine aminopeptidase rates after 24 h in both stations (paired test Wilcoxon, V-statistic = 0, *p* = 0.03125).

In the Ligurian Sea, leucine aminopeptidase activity ranged from 6.98 to 49.67 nMh^−1^_,_ displaying similar values in the first 6 h. After 24 h, a sharp increase of activity was observed in LC, and then it declined at 48 h. BC treatment inhibited the leucine aminopeptidase rate in the coastal and open stations after 24 h; this was statistically significant (paired test Wilcoxon, V-statistic = 0, *p* = 0.03125). In both locations, APase hydrolysis rates were lower in the BC amendments than in the controls (Fig. 3). BC treatments stayed somewhat stable and tended to decrease at 48 h. The decrease in APase after BC treatment was statistically significant in both Adriatic and Ligurian Seas after 24 h (paired test Wilcoxon, V-statistic = 0, *p* = 0.031). Due to logistic constraints, the prokaryotic carbon production was measured only during the Adriatic Sea experiments.

**Fig. 4:**
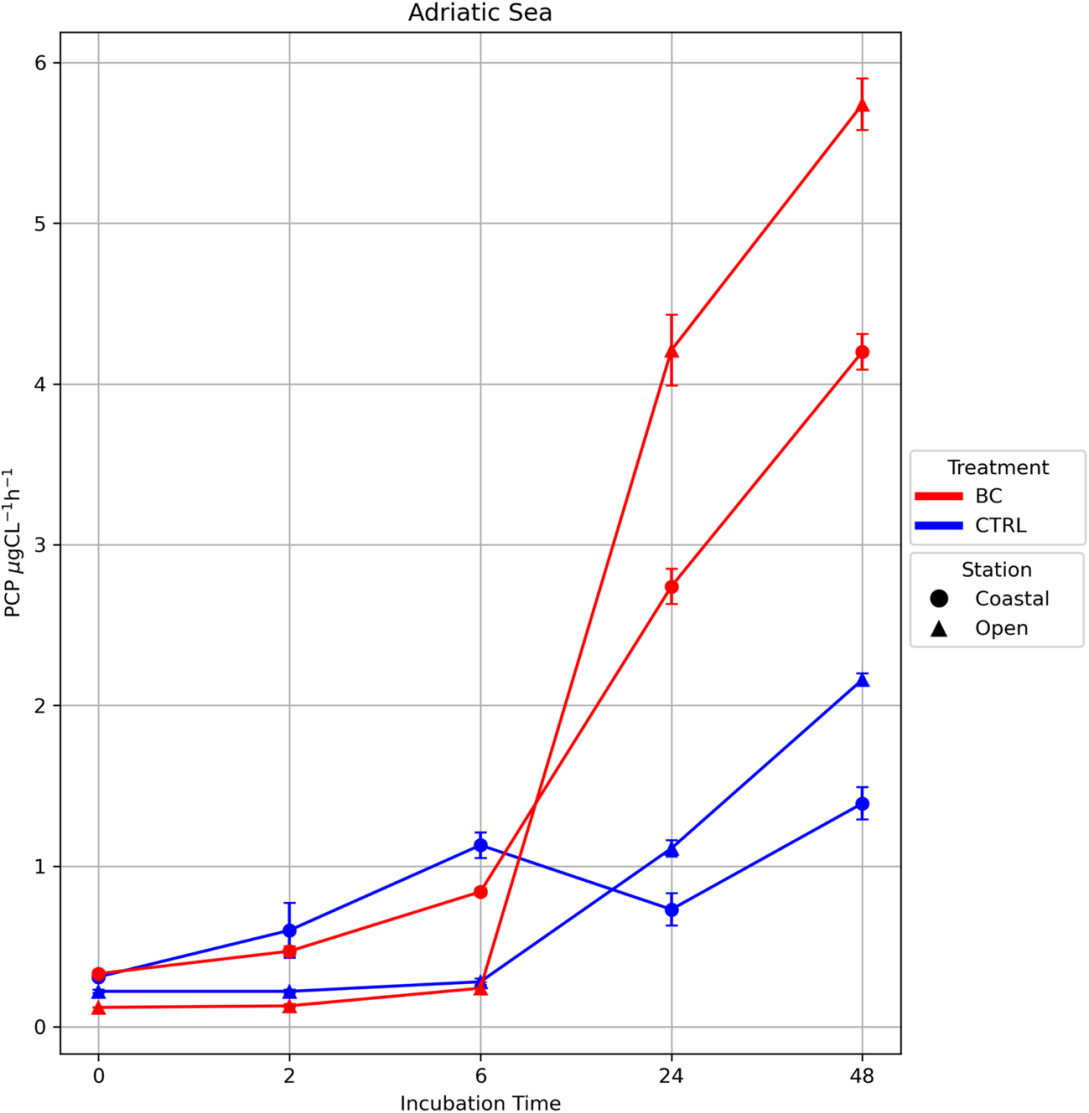
Prokaryotic carbon production (PCP) in the Adriatic Sea over time. Units are expressed as µgCL^−1^ h.^−1^. Color indicates treatment: black carbon (BC, red) and control (CTRL, blue). Shapes indicate stations: coastal (circle) and the open sea (triangle)

The prokaryotic carbon production in BC treatments sharply increased after 6 h and rose to 5.74 μgC L^−1^ h^−1^ in the open water station and 4.2 μgC L^−1^ h^−1^ in the coastal site (Fig. 4). PCP, in the control treatments, was 2.65 times lower in AO and 3.0 times lower in AC. In both control treatments, PCP grew at 48 h. The PCP increase in the BC treatments was statistically significant in both coastal and open stations (paired test Wilcoxon, V-statistic = 21, *p* = 0.03125).

### Prokaryotic diversity and community structure

**Fig. 5:**
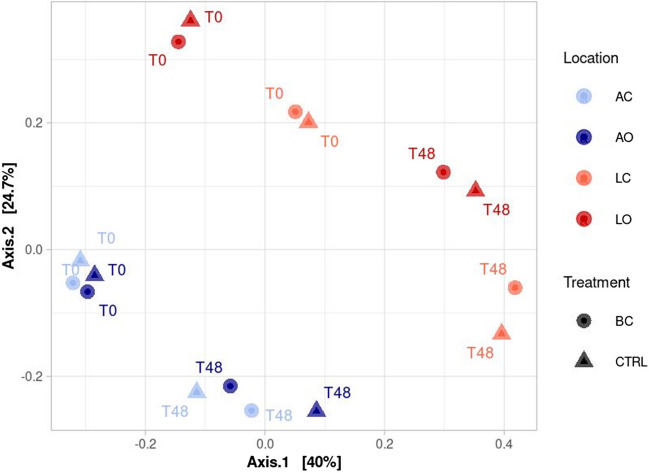
Principal component analysis (PCoA) based on Bray–Curtis dissimilarity matrix of 16S rDNA gene amplicon over time (0–48 h). Colors represent the different locations (cold colors, Adriatic Sea; warm colors, Ligurian Sea). Shapes indicate treatments: black carbon (BC, circle) and control (CTRL, triangle)

A total of 5,819,579 raw sequences were generated. The rarefaction curves (Fig. S3) reached the plateau, thus suggesting that the sequencing effort was enough to cover the microbial diversity. After the denoising step that included error correction and chimera removal, 2,760,073 reads were retained with an average of 172,504 ± 33,096 sequences per sample. The total number of ASVs across samples was 5083. Shannon’s diversity index and Pielou’s evenness index showed that the microbial communities in the Adriatic sea and the Ligurian sea were diverse (Fig. S4). Principal coordinates analysis (PCoA, Fig. 5) based on Bray–Curtis dissimilarity was performed to visualize dissimilarities in the microbial community structure across different treatments, locations, and incubation periods. The PCoA, in agreement with PERMANOVA grouped the samples by location (Adriatic vs Ligurian communities) and incubation time explaining a total variance of 64.7%.

According to PERMANOVA results, the location significantly contributed to the variability in microbial communities, explaining 41.98% of the total variance (*p* < 0.001). Additionally, incubation time also had a significant effect, accounting for 28.5% of the total variance (*p* < 0.001). However, the BC treatment did not significantly influence the microbial community structure, explaining only 1.51% of the total variance (*p* > 0.05). Overall, our results suggest that spatial and temporal variations (i.e., locations and incubation time) have a stronger influence on the structure of the microbial communities than the treatment itself.

**Fig. 6:**
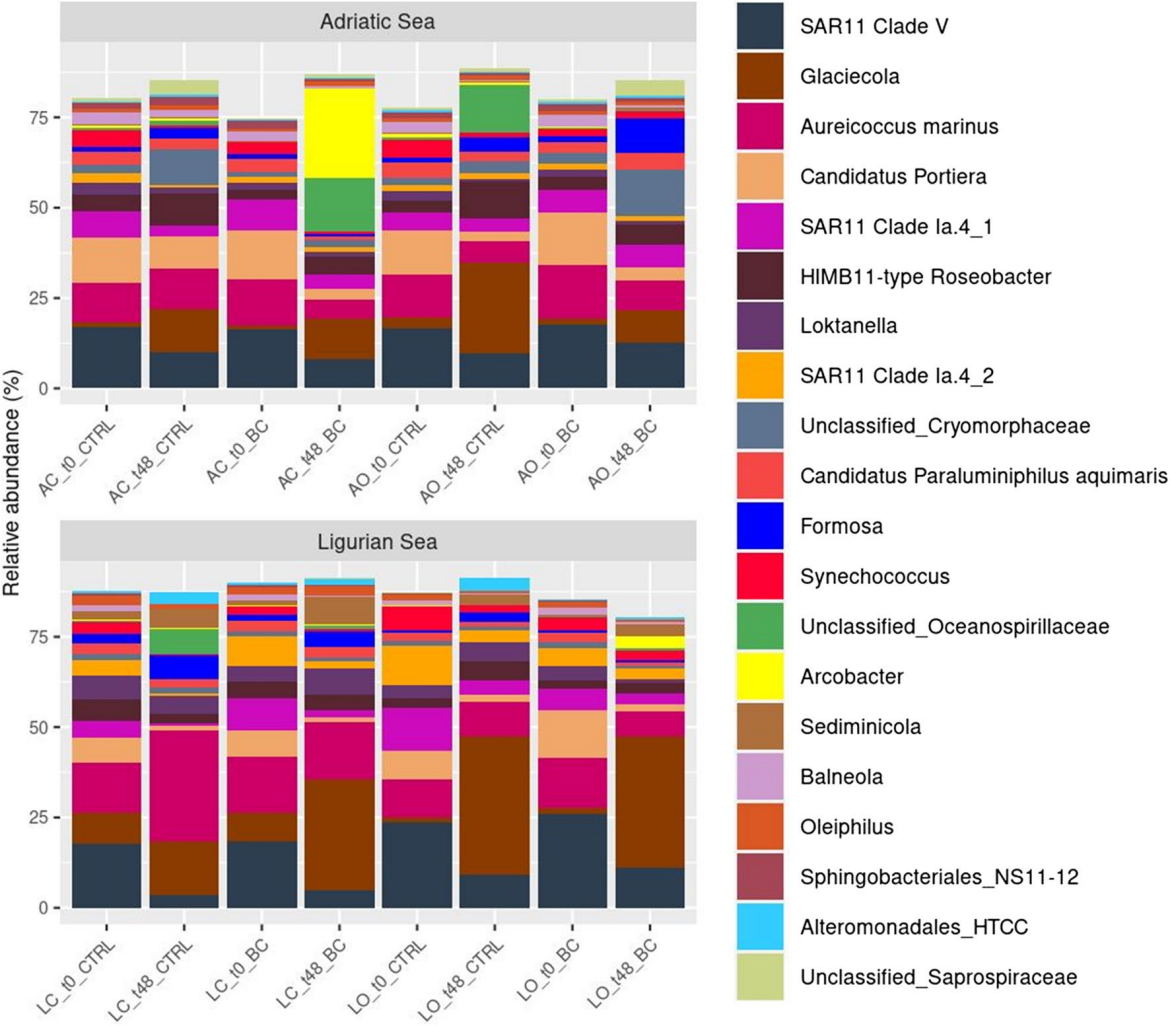
Taxonomic composition, relative abundance %, of the most abundant ASVs of the top 20 genera (agglomerated to genus level) in the treatments (BC, CTRL) at different stations (open, coastal) and incubation time (t0, t48) of the Adriatic (top panel) and Ligurian Sea (bottom panel)

Three phyla, Pseudomonadota (Proteobacteria, 67.75%), followed by Bacteroidota (25.22%) and Cyanobacteria (3.04%), accounted for over 80% of relative abundance in all samples. Cyanobacteria were more abundant, especially at T0. We focused our analysis on the top 20 most abundant genera that accounted for more than 80% of total relative abundance (Fig. 6, Family Barplot in Fig. S5). Across all the treatments and locations, *SAR 11 clade V* was the most abundant genus with higher relative abundance in BC than the CTRL (14.36 vs 13.4%) respectively, followed by *Glaciecola* and *Aureicoccus* marinus which presented an opposite distribution pattern and were more abundant in the CTRL (13.01%, 13.05%) than in the BC (12.43%, 11.68%) respectively. Over time, the relative abundance of *SAR11 clade V* decreased from 18.5 to 9.29%, while *Glaciecola* increased significantly from 4.23 to 21.21%, and *Aureicoccus marinus* stayed within the range of 12%. The experiments were run in the dark and this led to an overall decrease in relative abundance of *Synechococcus*. When comparing the Ligurian sea to the Adriatic sea, these three genera (*SAR11 clade V*, *Glaciecola*, and *Aureicoccus marinus*) were more abundant in the first basin (14.29%, 17.47%, and 14.51%, respectively) than in the latter basin (13.51%, 7.97%, 10.22% respectively).

*Arcobacter* was more abundant in BC treatment (3.59%, average relative abundance across the data set) than in the CTRL (0.48%, average relative abundance across the data set) where it was extremely rare, especially in the Adriatic at T48.

The indicator species analysis (Table 1) identified putative ASVs statistically associated with treatment and time across all the experiments. Specifically, by considering taxon abundance, *Arcobater*, *Pseudoalteromonas*, and *Pseudoalteromonas porphyrae* were statistically present with BC at T48. *Glaciecola* was consistently associated with both treatments at T48. The control treatment and the BC shared 7 taxa at T0, and BC presented 3 taxa that were statistically associated with it at T0.

**Table 1:** Indicator species analysis at the ASVs level of the whole dataset (*p* < 0.05). The factor is treatment (CTRL vs BC) over time (T0 and T48)

Treatment_Time	r.g (abundance)	*p*-value
BC-T0	- Candidatus Portiera (1 ASV)	0.0168
	- Unclassified_Pelagibacteraceae (2 ASVs)	0.0303; 0.0457
	- Alteromonadales_OM60 (2 ASVs)	0.0055; 0.0139
BC-CTRL-T0	- Unclassified_Alphaproteobacteria(1 ASV)	0.0043
	- Synechococcus (2 ASVs)	0.008; 0.026
	- Prochlorococcus (1 ASV)	0.0358
	- Loktanella (1 ASV)	0.0213
	- Unclassified_Pelagibacteraceae (2 ASVs)	0.0113; 0.042
	- Unclassified_Flavobacteriales (1 ASV)	0.0266
	- Octadecabacter (1 ASV)	0.0278
BC-CTRL-T48	- Glaciecola (1 ASV)	0.0328
BC-T48	- Pseudoalteromonas (1 ASV)	0.0278
	- Arcobacter (1 ASV)	0.0278
	- Pseudoalteromonas porphyrae (1 ASV)	0.04

## High-resolution FT-IR and AFM imaging of BC

We analyzed by FT-IR spectroscopy, BC samples from the Open Adriatic incubation at the end of the experiment. After the exposure to seawater, the average spectra of the BC changed.

**Fig. 7:**
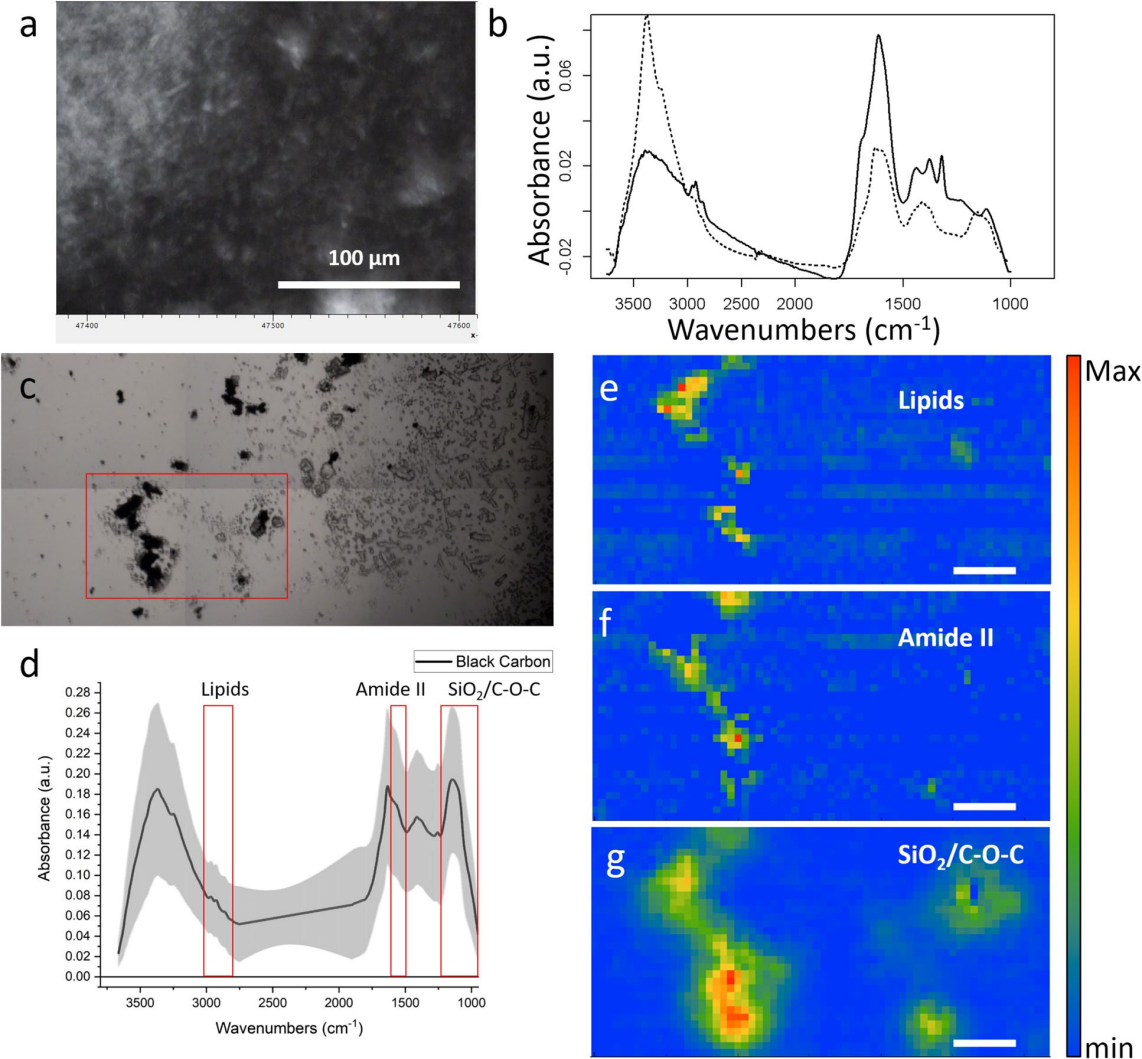
Composite panel of FT-IR spectroscopy and imaging. **a** Black carbon powder on paper filter. **b** Comparison of BC before (solid line, BC stock solution) and after (dotted line) long time exposure to seawater measured with ATR. **c** Optical image of the sample deposited onto the diamond cell. **d** Average spectrum of the BC material, the shading represents ± standard deviation, red boxes highlight the spectral region of interest used to the false color images in panels (**e**–**f**–**g**). **e**–**f**–**g** chemical distribution maps obtained by integrating the three bands highlighted in panel (**b**)

In Fig. 7b, a comparison of the BC is presented before (solid line, BC stock solution) and after incubation in seawater. From the spectra, it can be noticed a decrease in the overall aromatic and aliphatic carbon–carbon and carbon-hydrogen bonds in the 3000–2800 cm^−1^ range and the 1600 cm^−1^ band. The decrease of these signals was linked to an increase of the bands related to oxidized and hydrated species, as can be seen from the band in the ~ 3400 cm^−1^ which can be assigned to the -OH stretching mode. Since the BC was obtained from rice husk, a SiO2 band at ~ 1120 cm^−1^ is detectable.

BC underwent oxidation in seawater (Fig. 7c–g), due to the environment and possibly due to the microbial activities. In Fig. 7, panels e, f, and g show the results of the univariate analysis on one of the samples retrieved. It was possible to obtain IR false color images that portrayed the chemical distribution of an Amide II-like signal (1600–1480 cm^−1^) and the ones of CH3 and CH2 stretching vibrations (2800–3000 cm^−1^). Even if with this analysis, it is not possible to disentangle completely the signals of the organic material from those of the carbonaceous substrate and can be seen a good co-localization of the protein and lipid signals with the most of the carbon particles, in particular those in the left side.

**Fig. 8:**
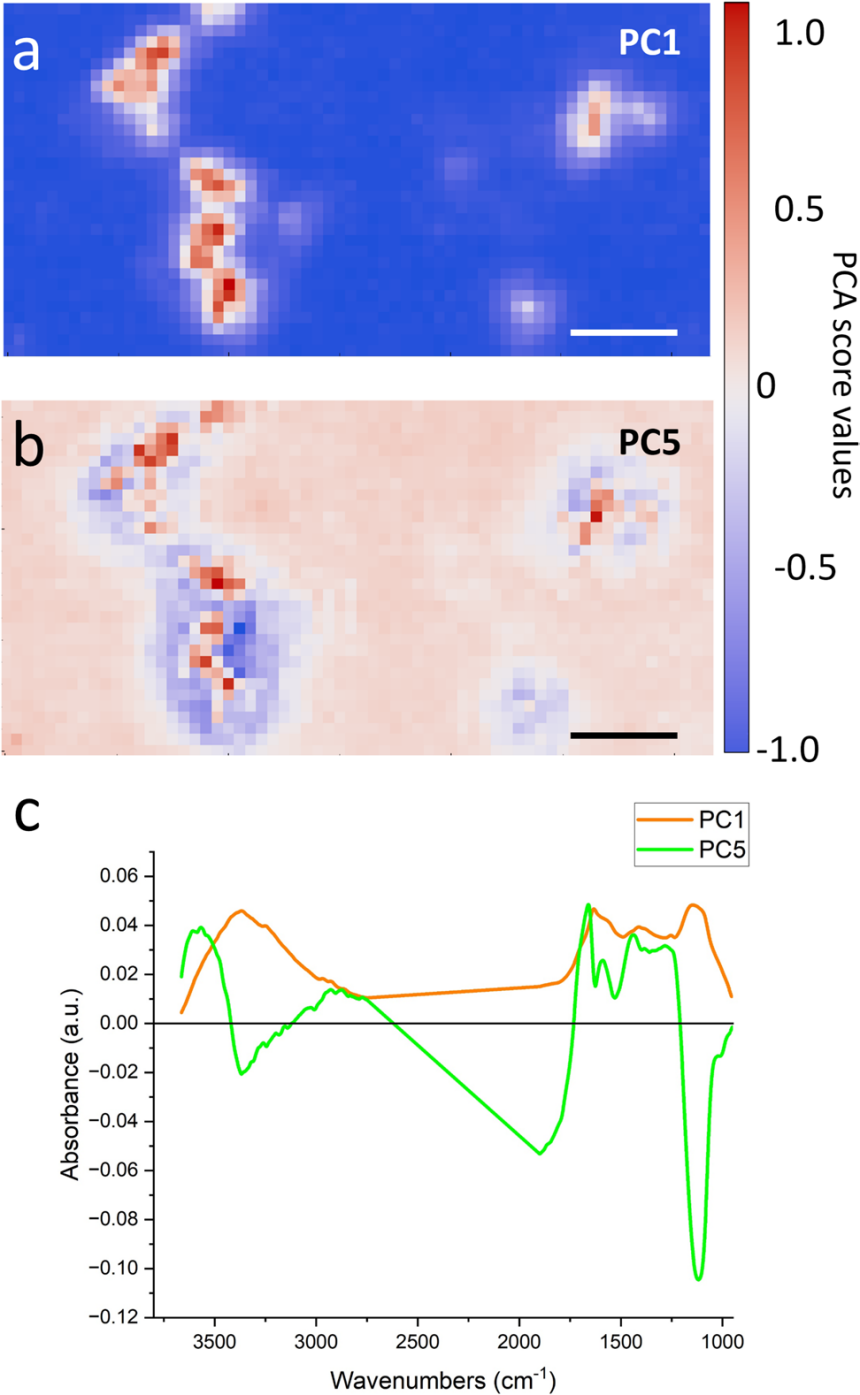
PCA analysis. **a** False color image obtained plotting the scores of PC1 that represent ~ 90% of the variance. **b** Image obtained plotting the scores of PC5, representing < 1% of the total variance. **c** Loading vectors of PC1 (orange) and PC5 (green) respectively

Therefore, in order to pinpoint the most representative spectral components characterizing the samples and highlight the presence of a bacterial component onto the BC, a principal component analysis, PCA, was performed (Fig. 8). With this analysis, we identified two interesting components: PC1 and PC5 (Fig. 8 c). PC1 contains the BC signals and is used mainly to visualize the areas of the sample where carbonaceous material is present and empty areas: this is the reason for this component to account for more than 90% of the total variance of the sample. PC5 instead accounts for less than 1% of the total; nevertheless, the signals present in his loading are similar to protein material. In particular, the main bands of interest of this component are the Amide I (1700–1600 cm^−1^) and Amide II (1600–1480 cm^−1^) bands, which can be attributed to the peptide bond in proteins. The stronger ones belonging to the carbonaceous materials would have hid these signals without the PCA analysis. Finally, by comparing the distribution of the red spots of PC1 and PC5, we can see that this protein-like signals strongly co-localize with the BC, as a preliminary analysis already pointed out.

**Fig. 9:**
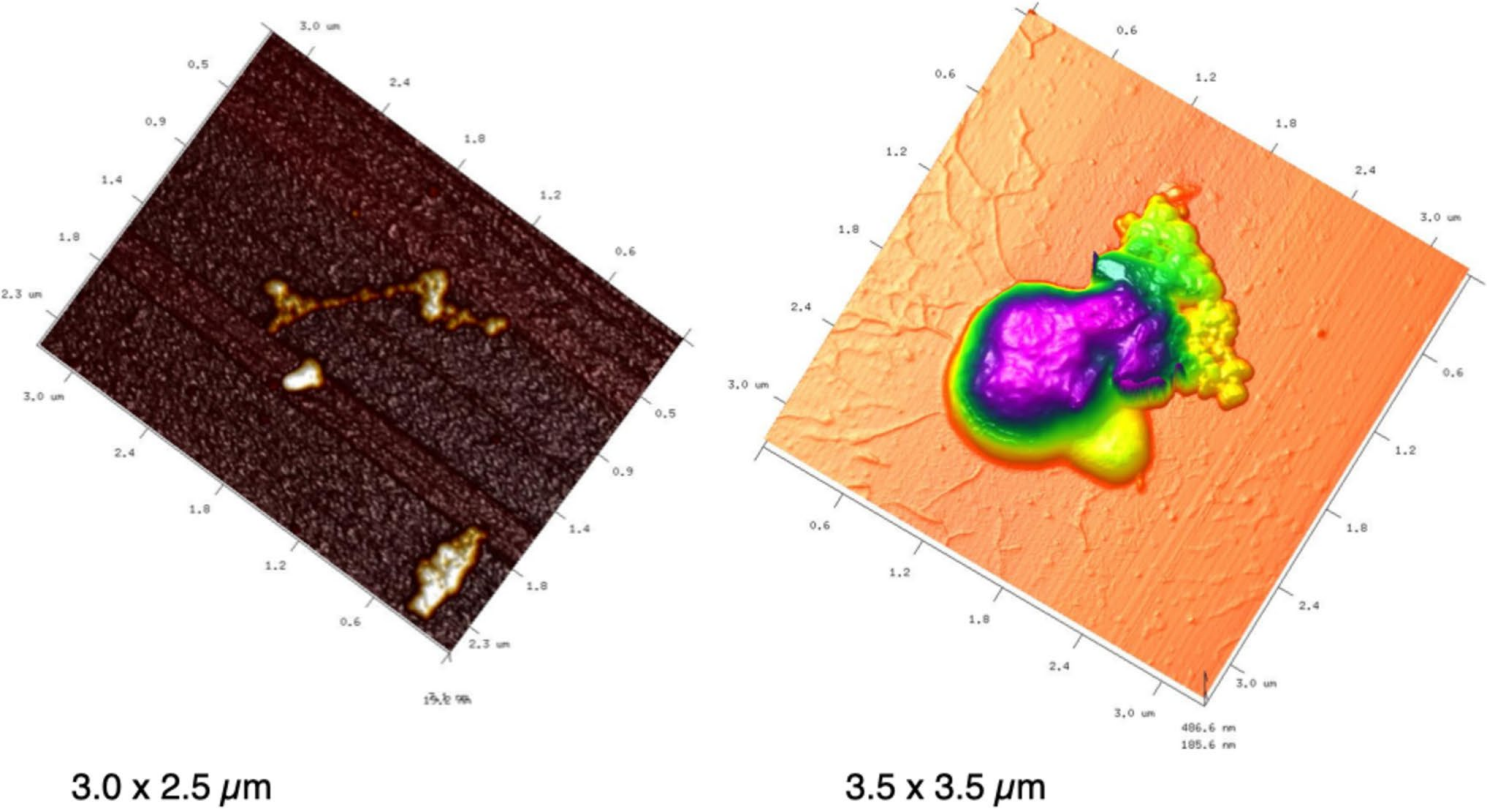
AFM topography image on BC stock solution (left panel) and clustered BC particles on seawater microbes (right panel). Pseudocolor code indicates height

BC stock solution showed the heterogeneity of size and shape of the BC powder, spanning from visible aggregates to smaller particles up to 100 nm (Fig. 9). Furthermore, during the pilot experiment, seawater samples showed that the smaller BC particles can be attached to marine microbes such as *Synechococcus* cells (that were identified due the size and shape at the AFM, see Malfatti et al. 2009) and heterotrophic prokaryotes. Samples were also imaged at LSCM, which showed prokaryotes and viruses being attached to larger BC particles (> 1 µm).

The microbial responses upon enrichment of anthropogenic-derived carbon, that we have observed during the incubation experiments, were able to impact the “natural” C biogeochemical cycle, thus influencing processes like organic matter degradation, C production, and nutrient turnover across both immediate, intermediate, and long timescales.

## Discussion

### BC affects microbial dynamics and activities

Once BC enters the ocean, it deconstructs the microbial biomass stocks, enhances prokaryotic carbon production, and possibly inhibits the organic matter processing activities. Upon BC addition, as expected, heterotrophic prokaryotes increased, whereas viruses decreased in number as reported by other studies (Mari et al. 2014; Malits et al. 2015; Cattaneo et al. 2010). This was in agreement with our first hypothesis that stated that the prokaryotic C production was enhanced in BC treatment. We can hypothesize that the microbial abundant increase is fueled by the BC release of some degradable DOC molecules (Martinot et al. 2023). It follows that based on the diverse BC origins, its chemical composition can be different, thus influencing microbial utilization. Furthermore, when considering the physical nature of these particles, BC is characterized by an heterogeneous size-continuum particle spectrum (Cattaneo et al. 2010 and Fig. 9, S10 pilot experiment, Hyp. 3). From the AFM and high-resolution laser scanning confocal microscope analyses run during the pilot experiment (see SI), we imaged two classes of microbes, the first “colonizing” the 3–10 µm BC particles (Fig. S10) and the second being “colonized” by the nm size BC particle fraction (Fig. 9). The colonization of these novel niches could have enhanced microbial abundance and prokaryotic carbon production in the BC treatments. On the other hand, being covered by BC nm-size particles could have stressed in situ the microbes possibly because BC can release toxic substances. BC is known to release polycyclic aromatic hydrocarbon compounds (Trilla-Prieto et al. 2021; Martinot et al. 2023), thus possibly influencing microbial community structure and gene expression. We did not find a solid shift in microbial community structure upon the BC amendment, so we cannot rule out the direct toxic BC effect. The pilot experiment (SI, Fig. S10) was run in anticipation to investigate the microscale structure of BC**,** which allowed us to isolate two bacterial strains that we identified by 16S rRNA gene Sanger sequencing as a *Pseudoalteromonas* (BCR strain) and *Alteromonas* (BCS strain). We operationally tested the toxicity of BC by assessing strain motility at the dark field microscope (Grossart et al. 2000). BCS was more susceptible to BC than BCR** (**Table S6). This result supports the idea that BC can affect the physiology and behavior of microbes also at about 240 × lower concentration (i.e., 0.1 mg L^−1^). BC could cause the death of some prokaryotic taxa, which in turn will release labile organic carbon, thus boosting the BC-resistant prokaryotes, and increasing the prokaryotic carbon production of the resistant taxa. Finally, BC can act as an attractor of DOC, thus strengthening even more the activity, colonization, and microbial carbon production on the particle itself, as observed also by others (Malits et al. 2015; Cattaneo et al. 2010, Mari et al. 2014). From the FT-IR analysis data (Fig. 7, 8S6, S7), BC particles at the end of the experiments in the Adriatic locations were enriched with small peptides and polyamino acids either deriving from the ambient DOC scavenging or from adsorbed viral particles, Hyp. 3. This mechanism can possibly even intensify the degree of microbial colonization on the BC particles, thus favoring the growth of the attached microbes.

Contrary to our hypothesis (Hyp. 1), the enzymatic activities were inhibited in the BC treatments even if the prokaryotic carbon production was enhanced. The low rates of leucine aminopeptidase could be explained by the BC scavenging activity of small peptides and polyamino acids (FT-IR analysis, Fig. 8 and SI). BC particles would have sequestered the proteinaceous substrates thus making them unavailable to the free-living prokaryotes. Alternatively, BC could have released inorganic N species that would have satisfied the N quota for microbes. We have found a decrease in total N species at the end of the BC treatments supporting the microbial growth (Table S5). Alkaline phosphatase rates were suppressed by the release of phosphate moieties from the BC particles; this P-enrichment was documented in this study (Table S5) and also found by others (Mari et al. 2019, Martinot et al. 2023). Besides, phosphate-rich molecules could have been trapped by BC particles (e.g., scavenging-like mechanism) thus making them unavailable to the prokaryotic cleavage.

In the future, it would be interesting to sample with higher frequency the free-living and the BC-particle attached prokaryotic communities to estimate the specific growth rates and the degradative activities and overall gene expression of pathways related to hydrocarbon degradation, enzyme expression, transporters, and stress-related genes. We still do not know if the BC-attached prokaryotes can grow or they die, thus enriching with fresh labile organic matter the surrounding environment, boosting prokaryotic C production for the free-living fraction. From a biochemical point of view, it is important to focus our future effort in characterizing the biological molecules that are scavenged on the BC particles in order to evaluate the potential activities, for instance, free-enzymes present on BC surfaces in cleaving the organic matter.

Phages are an integral part of the carbon biogeochemical cycle since they modulate population prokaryotic dynamics and community structure (Sime-Ngando 2014; Fuhrman et al. 2015). Viruses behave as colloids in the marine environment (Alldredge and Silver et al. 1988; Mojica and Brussaard, 2014), and can be adsorbed onto BC particles (Cattaneo et al. 2010 and Fig. S13). The free-viral load decreased over time and relieved prokaryotes from a strong top-down control. Marine viruses in the ocean display two main strategies: lytic and lysogenic (Payet and Suttle 2013; Ram et al. 2014). It has been shown that upon BC perturbation, there is a shift from lysogenic to lytic due to the stress in the host cells (Ram et al. 2018). A stressed prokaryotic host can influence the overall virion production and lead to a potential shift of viral replication (Sime-Ngando and Colombet 2009). The viruses to bacteria ratio, VBR, in our study was fluctuating over incubation time, highlighting the complexity of these interactions given the different hosts and viral cycles. It has been proposed that high VBR indicates a shift from lysogeny to lytic strategy (Ram et al. 2018), but our data are not conclusive. It would be interesting in the future to sample for viral community structure and set up viral production measurements to tease out the net effect of BC on viral population and on prokaryotes.

### Putative black carbon indicator taxa

Previous marine-based studies found that BC strongly affected the microbial community diversity and structure (Cattaneo et al. 2010; Cattaneo et al. 2010). It was remarkable to find that, in our work, the BC effect on the microbial communities was not highly significant, as we hypothesized. The lack of BC influence on microbial community diversity was also found in a nutrient-enriched marine sediment incubation experiment (Lu et al. 2021). We can explain our results by considering that BC influence on microbial communities could have been masked by the “bottle effect” (ZoBell and Anderson 1936). The “bottle effect,” the confinement of the microbes in a vessel, thus promoting copiotrophic and opportunistic taxa, has driven mostly the community shifts over time. Indeed, we found the presence of the genus *Glaciecola* significantly associated with all treatments over time. In previous BC studies, *Glaciecola* has been identified as a BC fingerprint taxa. *Glaciecola* belongs to the family of the Alteromonadaceae (Ivanova et al. 2004) that was very abundant in the experiments. This genus is widespread in the coastal ocean and it is characterized by its ability to degrade organic matter, especially complex polysaccharides (Bian et al. 2011; Mondal and Ohnishi 2020). Another aspect to consider in interpreting our results is that we have sampled the whole microbial community without fractionating the BC/particle-attached versus the free-living microbes. This might have skewed the signal of a BC effect on the microbial community structure. Finally, BC might have had a more pronounced effect on the physiology of the microbes by altering the gene expression not on the microbial communities since these locations are exposed to BC skyfall fluxes (Weinbauer et al. 2017; Pavese et al. 2012; Di Ianni et al. 2018). Nevertheless, it is rather intriguing the lack of a strong BC influence on the microbial community structure. It would be interesting in the future to test the community gene expression upon BC amendment to understand what are the adaptive strategies that coastal microbes can employ during this perturbation while sampling selectively for the free-living and the BC-attached microbes.

Even if the whole community did not shift substantially upon BC enrichment only, two genera emerged in BC treatments in all locations from the indicator species analysis, Hyp.2: *Arcobacter* and *Pseudoalteromonas*. Briefly, *Arcobacter* belongs to the Epsilonproteobacteria class and the Campylobactereaceae family (Vandamme et al. 1991). *Arcobacter* is a chemolithoheterotrophic and sulfur-oxidizing bacterium (Wirsen et al. 2002; Callbeck et al. 2019) found in marine coastal areas, seeps, salt marsh sediments, deep-sea hydrothermal vent chimney, hypersaline cyanobacterial mats, and redox interfaces (reference therein, Fera et al. 2004; Wirsen et al. 2002; Athen et al. 2021; Dick 2019; Patin et al. 2021). Moreover, it has been found also in stratified environments characterized by high sulfide and low oxygen concentration (Callbeck et al. 2019), possibly also BC particles. From the genome comparative analysis, *Arcobacter* has metal resistance and antibiotic resistance genes (Buzzanca et al. 2023; Fanelli et al. 2019). Overall, this genus can adapt in highly diverse microenvironments, given its genomic versatility (Buzzanca et al. 2023). The second genus found in the indicator species analysis and isolated in the pilot experiment was *Pseudoalteromonas*. It belongs to the Gammaproteobacteria class and Pseudoalteromonadaceae family (Gauthier et al. 1995; Ivanova et al. 2004). This genus has been frequently isolated from microalgae and detrital particles such as marine snow. It presents versatile metabolisms and can express a great diversity of hydrolytic enzymatic activities, secondary metabolites with antimicrobial actions. It is motile and can form biofilm (Bowman 2007; Munn 2020). From genomic comparative analysis, *Pseudoalteromonas* has siderophores genes, specific carbohydrate transporters, and phage-like contractile injection systems (Sonnenberg and Haugen 2021; Alker et al. 2021). Being able to scavenge iron, form biofilm, fight off competitors with secondary metabolites, and inject toxin by contractile secretion systems allow *Pseudoalteromonas* to thrive also on particles such as BC. Both genera are known to be able to withstand pH drop up to 4–5 units (Park 2005; Cervenka 2007; Wesley and Miller, 2010; Yan et al. 2016), and they are able to metabolize a range of organic carbon sources, including aromatic compounds and hydrocarbons (Evans et al. 2018; Zan et al. 2021).

It was reported that BC can acidify the water by causing a pH drop of 0.1 units at approximately 1 mg L^−1^ (Weinbauer et al. 2017). We did not measure the pH in our experiments, but we can hypothesize that there was a change in seawater chemistry upon BC addition. This could have affected the microbial community gene expression (Logue and Lindström, 2008) thus influencing certain taxa’s prevalence. Thus, pH shifts could favor marine microbes capable of acclimatizing to an increase in H + in the short and long term (Saidi et al. 2023; Joint et al. 2011).

We like to hypothesize that the BC amendment can create microniches where chemolithoheterotroph, sulfur-oxidizing, lower pH resistant, and hydrocarbon-degrading microbes can thrive in the lit and well-oxygenated ocean water. BC is rich in sulfur and nitrogen compounds that can leak out from the particle phase and become dissolved black carbon (Wagner et al. 2018; Luo et al. 2019).

BC particles scavenge viruses and possibly other molecules such as proteins including free-enzymes. According to the size of the BC particles, we hypothesize that the BC structuring effect has a role reversal according to the size category, either BC creates a novel substrate for BC-resistant taxa or nm-size particles can deposit onto the microbial surfaces, thus concentrating inactivated viruses and proteins potentially fuelling the microbial metabolisms but also can cause a localized release of toxic substances (e.g., hydrocarbons and heavy metals).

Our study aligns with the broader framework of marine microbial ecology and BC research, highlighting how BC-mediated shift in seawater can create distinct microniches that modulate microbial activity and potentially interspecies interactions. Specifically, the emergence of certain taxa in response to the BC amendment over time highlights its ability to reshape ecological dynamics, with significant implications for coastal carbon cycling and pollutant fate.

In sum, future studies should focus on long-term monitoring of BC effect on microbial dynamics and structure aiming at better understanding BC contribution to the C biogeochemical cycle in the ocean. A holistic approach is therefore urgent in order to couple next-generation transcriptomic and metabolomic with high-resolution organic matter fingerprint and chemical characterization of microbial by-products of BC degradation and their effect on prokaryotes.

## Conclusion

In conclusion, our study revealed the multifaceted impact of black carbon on microbial functioning of marine ecosystems. Specifically, BC impacts significantly the microbial dynamics by enhancing heterotrophic prokaryote abundance and carbon production and decreasing viral loads and degradative enzymatic activities in the absence of primary production. At the microbial community levels, over time, BC-treated samples were characterized by the prevalence of *Arcobacter* and *Pseudoalteromonas*, and possibly, some taxa have been killed. These findings start to depict the complex and variable relationships between BC and marine microbes, thus calling for more experiments focusing on microbial gene expression of BC-related metabolisms of free-living and BC-attached communities. Deepening our understanding of the ecological implication of BC deposition in the microbial ocean will help better constrain the overall C pumps: biological, solubility, and MCPs (microbial carbon pump; Jiao et al. 2011) in the presence of anthropogenic-derived BC particles.

## Supplementary Information

Below is the link to the electronic supplementary material.

Supplementary Material 1 (PDF 4.07 MB)

## Data Availability

Fastq sequence files are available at the National Center for Biotechnology Information in Sequence Read Archive (SRA) repository (accession number: BioProject PRJNA1002595). https://www.ncbi.nlm.nih.gov/sra/PRJNA1002595
