# Supplementary material for: Black carbon structuring marine microbial activities and interactions: a micro- to macro-scale interrogation
Source: Environ Sci Pollut Res Int. 2025 Jun 13;32(26):15657–75. doi: 10.1007/s11356-025-36603-0 (PMC12238146; doi:10.1007/s11356-025-36603-0)
Supplement: Supplementary file 1 — Supplementary Material 1 (PDF 4.07 MB) [file 11356_2025_36603_MOESM1_ESM.pdf]

## Supplementary Information (SI) to:

# Black Carbon structuring marine microbial activities and interactions: a micro- to macro-scale interrogation

*Amira Saidi[1,2], Luca Zoccarato[3], Giovanni Birarda[5], Xavier Mari[6], Markus Weinbauer[7], Lisa Vaccari[5], Mauro Celussi[1], Francesca Malfatti[1,7*]*

[1] Oceanography Section, National Institute of Oceanography and Applied Geophysics – OGS, Via Auguste Piccard, 54, 34151, Trieste, Italy
[2] Department of Environmental Sciences, Informatics and Statistics Ca'Foscari Università di Venezia, Via Torino 155, 30172 Mestre Venezia, Italy
[3] Institute of Computational Biology, University of Natural Resources and Life Sciences (BOKU), Muthgasse 18, Vienna 1190 ; Core Facility Bioinformatics, University of Natural Resources and Life Sciences (BOKU), Muthgasse 18, Vienna 1190, Austria
[4] Elettra – Sincrotrone Trieste, Strada Statale 14 - km 163,5 in AREA Science Park, 34149 Basovizza, Trieste, Italy
[5] Mediterranean Institute of Oceanography - MIO, Marine Environment Chemistry (CEM), Aix Marseille Université, Université de Toulon, CNRS, IRD, 163 avenue de Luminy - Bâtiment Méditerranée 13288 Marseille, France
[6] Laboratoire d'Océanographie de Villefranche, LOV Institut de la Mer de Villefranche, IMEV 181 Chemin du Lazaret 06230 Villefranche-sur-Mer (France) Sorbonne Universités, UPMC, Université Paris 06, CNRS
[7] Life Sciences Department, Università Degli Studi Di Trieste, Via Fleming 22, 34127, Trieste, Italy

*: Corresponding author, fmalfatti@units.it

***Table S1*** Sea, Sampling stations, study ID, coordinates and historical names, depths and date

| Basin | Station | Study ID | Historical names | Latitude [°N] | Longitude [°E] | Sampling Depth [m] | Date |
|---|---|---|---|---|---|---|---|
| Adriatic Sea | Open-Sea | AO | LTER-C1 | 45.42299 | 13.423600 | 1 | 21 Sep 2015 |
| | Coastal | AC | Brojenca | 45.741108 | 13.668963 | 1 | 22 Sep 2015 |
| Ligurian Sea | Open-Sea | LO | Point B | 43.68333 | 7.31667 | 1 | 9 Jun 2015 |
| | Coastal | LC | Ponton | 43.696328 | 7.307562 | 1 | 10 Jun 2015 |

***Table S2*** Water column data at different locations of Open and Coastal Adriatic and Ligurian Sea: HP = Heterotrophic Prokaryotic abundance; SYN = *Synechococccus* abundance; PCP = Prokaryotic Carbon Production; Leu-AMA = leucine aminopeptidase activity; APase = alkaline phosphatase activity

| | Location ID | Treatment | Time | HP | | SYN | | PCP | | Viruses | | Leu-AMA | | APase | |
|---|---|---|---|---|---|---|---|---|---|---|---|---|---|---|---|
| | | | | cells/L | stdev | cells/L | stdev | µgC/L/h | stdev | Vlps/L | stdev | nM/h | stdev | nM/h | stdev |
| Adriatic | [illegible] | CTRL | 0 | 7.89E+08 | 1.95E+07 | 5.92E+07 | 1.32E+06 | 0.22 | 0.01 | 6.73E+09 | 9.76E+08 | 44.23 | 2.95 | 20.7 | 0.77 |
| | | CTRL | 2 | 7.59E+08 | 1.17E+07 | 6.26E+07 | 2.70E+05 | 0.22 | 0.01 | 6.65E+09 | 1.61E+08 | 18.99 | 3.82 | 19.49 | 0.42 |
| | | CTRL | 6 | 7.52E+08 | 1.77E+07 | 6.24E+07 | 2.27E+06 | 0.28 | 0.02 | 6.78E+09 | 5.46E+08 | 75.73 | 7.87 | 21.81 | 0.62 |
| | | CTRL | 24 | 1.05E+09 | 3.05E+07 | 6.08E+07 | 2.37E+06 | 1.11 | 0.05 | 6.47E+09 | 4.72E+08 | 253.25 | 3 | 27.15 | 0.34 |
| | | CTRL | 48 | 1.49E+09 | 5.00E+07 | 4.66E+07 | 1.14E+06 | 2.16 | 0.04 | 6.77E+09 | 1.45E+08 | 347.7 | 7.02 | 35.92 | 1.5 |
| | | BC | 0 | 6.80E+08 | 5.25E+07 | 5.87E+07 | 4.50E+06 | 0.12 | 0 | 5.96E+09 | 1.57E+09 | 36.32 | 4.9 | 16.78 | 0.36 |
| | | BC | 2 | 6.92E+08 | 2.29E+07 | 5.99E+07 | 4.57E+05 | 0.13 | 0.01 | 6.02E+09 | 2.86E+08 | 30.02 | 14.44 | 15.54 | 0.48 |
| | | BC | 6 | 6.40E+08 | 4.45E+07 | 5.97E+07 | 7.07E+05 | 0.24 | 0.04 | 5.35E+09 | 3.84E+08 | 61.4 | 5.67 | 16.81 | 0.39 |
| | | BC | 24 | 1.63E+09 | 5.50E+07 | 5.71E+07 | 1.26E+06 | 4.21 | 0.22 | 5.16E+09 | 8.74E+07 | 248.29 | 6.19 | 16.19 | 0.63 |
| | | BC | 48 | 1.99E+09 | 3.25E+07 | 3.53E+07 | 6.28E+05 | 5.74 | 0.16 | 5.26E+09 | 2.26E+08 | 219.59 | 0.75 | 10.95 | 0.5 |
| | [illegible] | CTRL | 0 | 5.74E+08 | 2.76E+07 | 4.46E+07 | 1.10E+06 | 0.31 | 0 | 5.18E+09 | 8.34E+07 | 70.82 | 1.15 | 19.62 | 0.16 |
| | | CTRL | 2 | 5.63E+08 | 1.80E+07 | 4.30E+07 | 3.81E+05 | 0.6 | 0.17 | 4.98E+09 | 1.96E+08 | 78 | 1.78 | 19.23 | 0.53 |
| | | CTRL | 6 | 7.35E+08 | 1.99E+07 | 4.43E+07 | 8.65E+05 | 1.13 | 0.08 | 6.35E+09 | 2.34E+08 | 116.18 | 4.29 | 17.85 | 0.23 |
| | | CTRL | 24 | 1.78E+09 | 4.80E+07 | 4.05E+07 | 7.63E+05 | 0.73 | 0.1 | 6.82E+09 | 3.18E+08 | 205.94 | 4.13 | 33.49 | 0.64 |
| | | CTRL | 48 | 1.82E+09 | 3.36E+07 | 2.35E+07 | 1.43E+06 | 1.39 | 0.1 | 6.47E+09 | 1.78E+08 | 176.97 | 6.54 | 57.72 | 6.18 |
| | | BC | 0 | 5.12E+08 | 1.43E+07 | 4.33E+07 | 8.34E+05 | 0.33 | 0.02 | 4.12E+09 | 7.85E+07 | 61.59 | 1.36 | 13.6 | 0.35 |
| | | BC | 2 | 5.06E+08 | 3.30E+07 | 4.31E+07 | 2.73E+05 | 0.47 | 0.03 | 3.99E+09 | 1.62E+08 | 65.14 | 2.85 | 13.23 | 1.41 |
| | | BC | 6 | 6.59E+08 | 4.56E+07 | 4.22E+07 | 4.18E+05 | 0.84 | 0.02 | 5.43E+09 | 4.77E+08 | 78.87 | 1.66 | 12.88 | 0.45 |
| | | BC | 24 | 1.91E+09 | 8.22E+07 | 4.02E+07 | 7.96E+05 | 2.74 | 0.11 | 5.48E+09 | 5.40E+08 | 145.11 | 1.53 | 8.78 | 0.5 |
| | | BC | 48 | 2.27E+09 | 9.20E+07 | 2.35E+07 | 9.77E+05 | 4.2 | 0.11 | 4.99E+09 | 2.55E+08 | 127.08 | 2.88 | 8.62 | 1.35 |
| Ligurian | [illegible] | CTRL | 0 | 3.64E+08 | 2.77E+07 | 1.32E+07 | 2.65E+06 | | | 3.96E+09 | 5.48E+08 | 6.98 | 1.12 | 11.21 | 1.13 |
| | | CTRL | 2 | 3.54E+08 | 6.22E+07 | 1.61E+07 | 3.63E+05 | | | 5.20E+09 | 3.25E+08 | 12.19 | 5.28 | 11.27 | 0.47 |
| | | CTRL | 6 | 3.02E+08 | 1.01E+07 | 1.60E+07 | 2.08E+05 | | | 6.81E+09 | 5.27E+08 | 10.12 | 6.18 | 8.77 | 0.1 |
| | | CTRL | 24 | 4.48E+08 | 3.42E+07 | 1.14E+07 | 8.02E+05 | | | 4.31E+09 | 5.23E+08 | 35.72 | 4.08 | 21.55 | 1.23 |
| | | CTRL | 48 | 8.07E+08 | 4.01E+07 | 1.91E+06 | 9.71E+04 | | | 3.89E+09 | 1.56E+08 | 99.95 | 7.75 | 42.88 | 3.31 |
| | | BC | 0 | 2.72E+08 | 1.80E+06 | 1.36E+07 | 2.57E+06 | | | 3.00E+09 | 5.25E+08 | 13.09 | 2.25 | 9.21 | 0.18 |
| | | BC | 2 | 2.27E+08 | 4.56E+06 | 1.63E+07 | 1.26E+05 | | | 3.84E+09 | 1.29E+08 | 11.17 | 4.92 | 10.37 | 1.13 |
| | | BC | 6 | 2.38E+08 | 1.20E+07 | 1.65E+07 | 5.65E+05 | | | 4.19E+09 | 1.92E+08 | 15.54 | 2.17 | 9.14 | 0.42 |
| | | BC | 24 | 2.82E+08 | 1.60E+07 | 1.29E+07 | 6.62E+05 | | | 3.57E+09 | 2.38E+08 | 31.05 | 4.16 | 8.11 | 0.33 |
| | | BC | 48 | 6.70E+08 | 2.35E+07 | 2.64E+06 | 1.71E+06 | | | 3.53E+09 | 2.45E+08 | 80.11 | 8.12 | 9.54 | 1.14 |
| | [illegible] | CTRL | 0 | 3.81E+08 | 1.72E+07 | 1.17E+07 | 1.23E+05 | | | 2.12E+09 | 1.25E+08 | 43.42 | 0.48 | 37.3 | 2 |
| | | CTRL | 2 | 4.46E+08 | 3.24E+07 | 1.15E+07 | 7.02E+05 | | | 4.11E+09 | 5.58E+08 | 45.7 | 2.96 | 33.9 | 5.4 |
| | | CTRL | 6 | 5.41E+08 | 8.04E+06 | 1.29E+07 | 3.82E+05 | | | 3.57E+09 | 2.66E+07 | 85.11 | 3.21 | 42.4 | 5.3 |
| | | CTRL | 24 | 1.26E+09 | 1.39E+08 | 1.07E+07 | 1.72E+05 | | | 3.57E+09 | 1.69E+08 | 611.05 | 23.93 | 45.4 | 11.5 |
| | | CTRL | 48 | 1.53E+09 | 1.06E+08 | 6.09E+06 | 4.86E+04 | | | 4.57E+09 | 1.04E+09 | 546.03 | 19.57 | 41.2 | 1.2 |
| | | BC | 0 | 3.49E+08 | 5.77E+06 | 1.08E+07 | 7.56E+05 | | | 1.85E+09 | 6.72E+08 | 49.67 | 1.63 | 23.9 | 0.9 |
| | | BC | 2 | 3.61E+08 | 2.97E+07 | 1.05E+07 | 7.42E+05 | | | 2.70E+09 | 3.59E+08 | 38.43 | 0.78 | 25.9 | 10.5 |
| | | BC | 6 | 4.16E+08 | 2.36E+07 | 1.21E+07 | 5.32E+05 | | | 2.53E+09 | 1.22E+08 | 77.33 | 3.12 | 30.6 | 10.2 |
| | | BC | 24 | 1.52E+09 | 3.58E+07 | 1.09E+07 | 3.89E+05 | | | 3.02E+09 | 8.00E+07 | 277.74 | 11.95 | 25 | 8.8 |
| | | BC | 48 | 2.10E+09 | 9.07E+07 | 6.16E+06 | 8.59E+05 | | | 3.96E+09 | 8.86E+08 | 323.34 | 75.7 | 24.4 | 1.2 |

***Table S3*** Attached heterotrophic prokaryotes (HP) and attached *Synechococcus* (SYN) abundance and percentage

| | Location ID | Time | HP-attached | | % HP-attached | SYN-attached | | % SYN-attached |
|---|---|---|---|---|---|---|---|---|
| | | | cells/L | stdev | | cells/L | stdev | |
| Adriatic | Open | 2 | 4.52E+07 | 6.96E+06 | 6.53 | 7.29E+05 | 2.42E+05 | 1.22 |
| | | 6 | 3.15E+07 | 5.42E+06 | 4.92 | 4.39E+05 | 2.24E+05 | 0.74 |
| | | 24 | 8.31E+07 | 2.73E+07 | 5.11 | 8.95E+05 | 4.36E+05 | 1.57 |
| | | 48 | 5.47E+07 | 0 | 2.75 | 7.86E+05 | 2.74E+05 | 2.23 |
| | Coastal | 2 | 4.08E+07 | 1.68E+07 | 8.07 | 6.20E+05 | 2.51E+05 | 1.44 |
| | | 6 | 2.54E+07 | 5.19E+06 | 3.86 | 9.23E+05 | 3.37E+05 | 2.19 |
| | | 24 | 6.61E+07 | 4.60E+06 | 3.46 | 5.76E+05 | 1.67E+05 | 1.43 |
| | | 48 | 7.84E+07 | 1.54E+07 | 3.45 | 4.76E+05 | 1.19E+05 | 2.03 |
| Ligurian | Open | 2 | 6.34E+07 | 9.63E+06 | 27.92 | | | |
| | | 6 | 4.52E+07 | 5.91E+06 | 19.04 | | | |
| | | 24 | 3.36E+07 | 9.12E+06 | 11.92 | | | |
| | | 48 | 5.92E+07 | 7.59E+06 | 8.84 | | | |
| | Coastal | 2 | 1.81E+07 | 1.56E+06 | 5 | | | |
| | | 6 | 6.23E+07 | 0 | 14.97 | | | |
| | | 24 | 8.82E+07 | 2.13E+07 | 5.78 | | | |
| | | 48 | 1.09E+08 | 1.41E+07 | 5.21 | | | |

***Table S4*** Differences in viral abundance at T0 in all experiments

| | **Location ID** | **Difference** |
|---|---|---|
| **Adriatic Sea** | Open | 0.77 |
| | Coastal | 1.06 |
| **Ligurian Sea** | Open | 0.96 |
| | Coastal | 0.27 |

***Table S5*** Phosphate (P-PO4), nitrite (N-NO2), nitrate (N-NO3) and ammonia (N-NH4) concentrations in the experiments before and after BC amendment in the Ligurian and Adriatic experiments

| Bassin | Location ID | Treatment | Incubation Time | P-PO4 | N-NO2 | N-NO3 | N-NH4 |
|---|---|---|---|---|---|---|---|
| Adriatic | Coastal | CTRL | T0 | 0.06 | 0.08 | 3.01 | 9.41 |
| | | | T48 | 0.04 | 0.09 | 3.61 | 0.6 |
| | | BC | T0 | 0.47 | 0.09 | 2.90 | 1.76 |
| | | | T48 | 0.43 | 0.12 | 2.55 | 0.32 |
| | Open | CTRL | T0 | 0.03 | 0.06 | 2.02 | 1.04 |
| | | | T48 | 0.02 | 0.07 | 2.43 | 0.94 |
| | | BC | T0 | 0.41 | 0.06 | 1.78 | 3.92 |
| | | | T48 | 0.69 | 0.19 | 1.94 | 0.67 |
| Ligurian | Coastal | CTRL | T0 | 0.02 | 0.03 | 1.82 | — |
| | | | T48 | 0.03 | 0.03 | 1.53 | — |
| | | BC | T0 | 0.53 | 0.05 | 1.35 | — |
| | | | T48 | 0.35 | 0.04 | 0.92 | — |
| | Open | CTRL | T0 | | | | — |
| | | | T48 | 0.03 | 0.01 | 1.04 | — |
| | | BC | T0 | | | | — |
| | | | T48 | 0.54 | 0.02 | 0.82 | — |

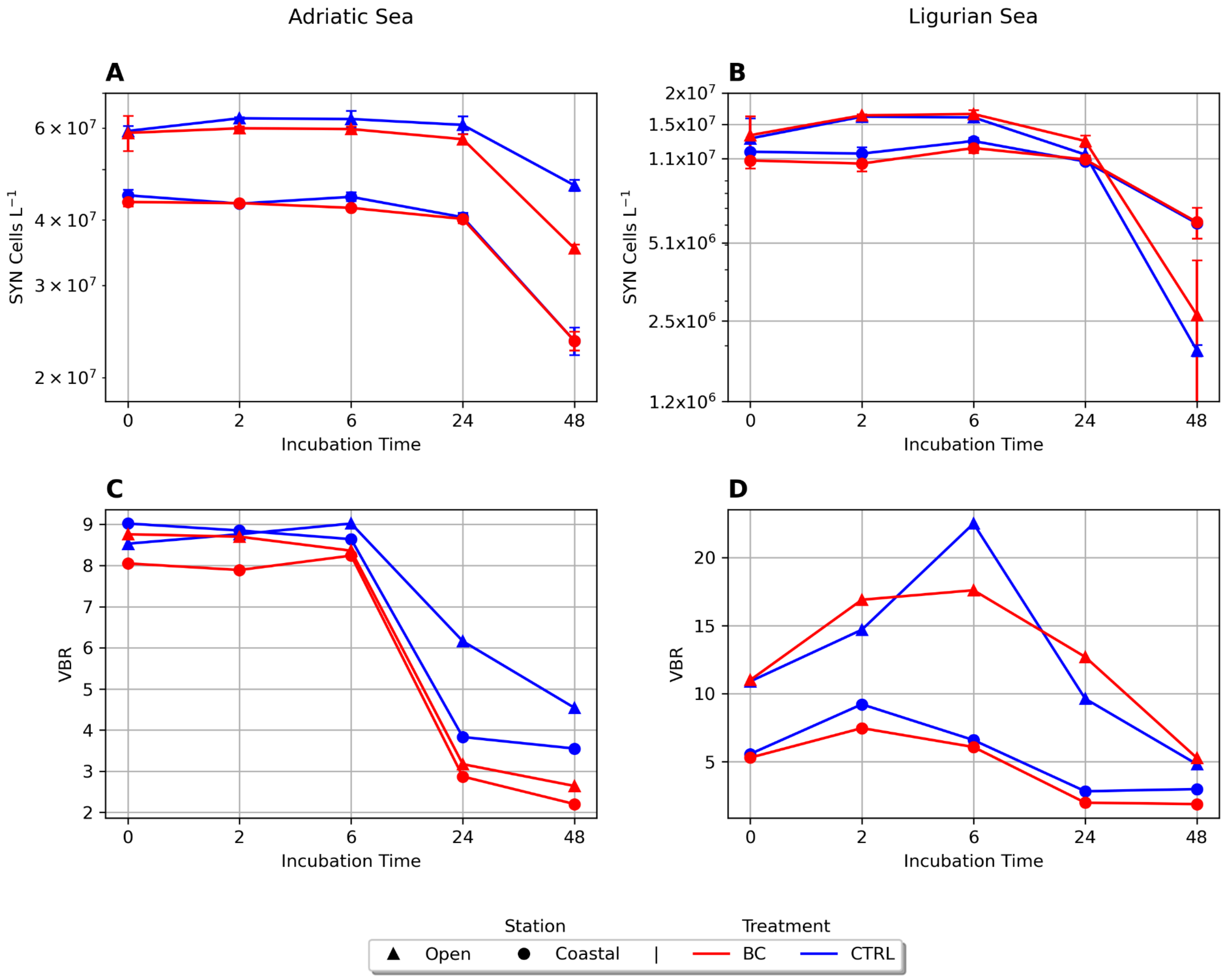


***Fig. S1*** *Synechococcus* abundance (SYN cells $L^{-1}$) and virus to bacteria ratio (VBR) over incubation time in the different treatments. Color indicates treatment: black carbon (BC, red) and control (CTRL, blue). Shapes indicate stations: coastal (circle) and the open sea (triangle)

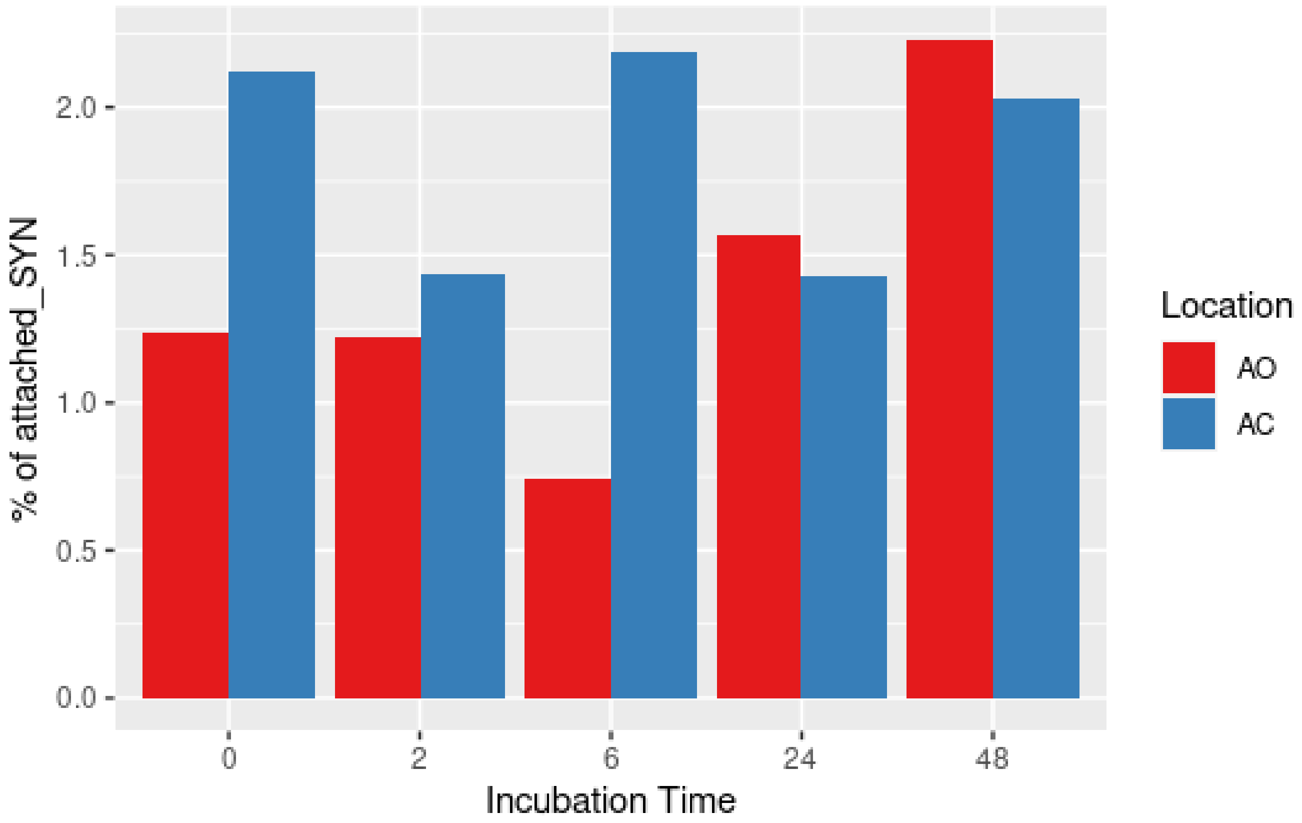

***Fig. S2*** Percentage of attached *Synechoccoccus* cells onto BC in the Adriatic Sea in both Location (Open: red and Coastal: Blue) during incubation time

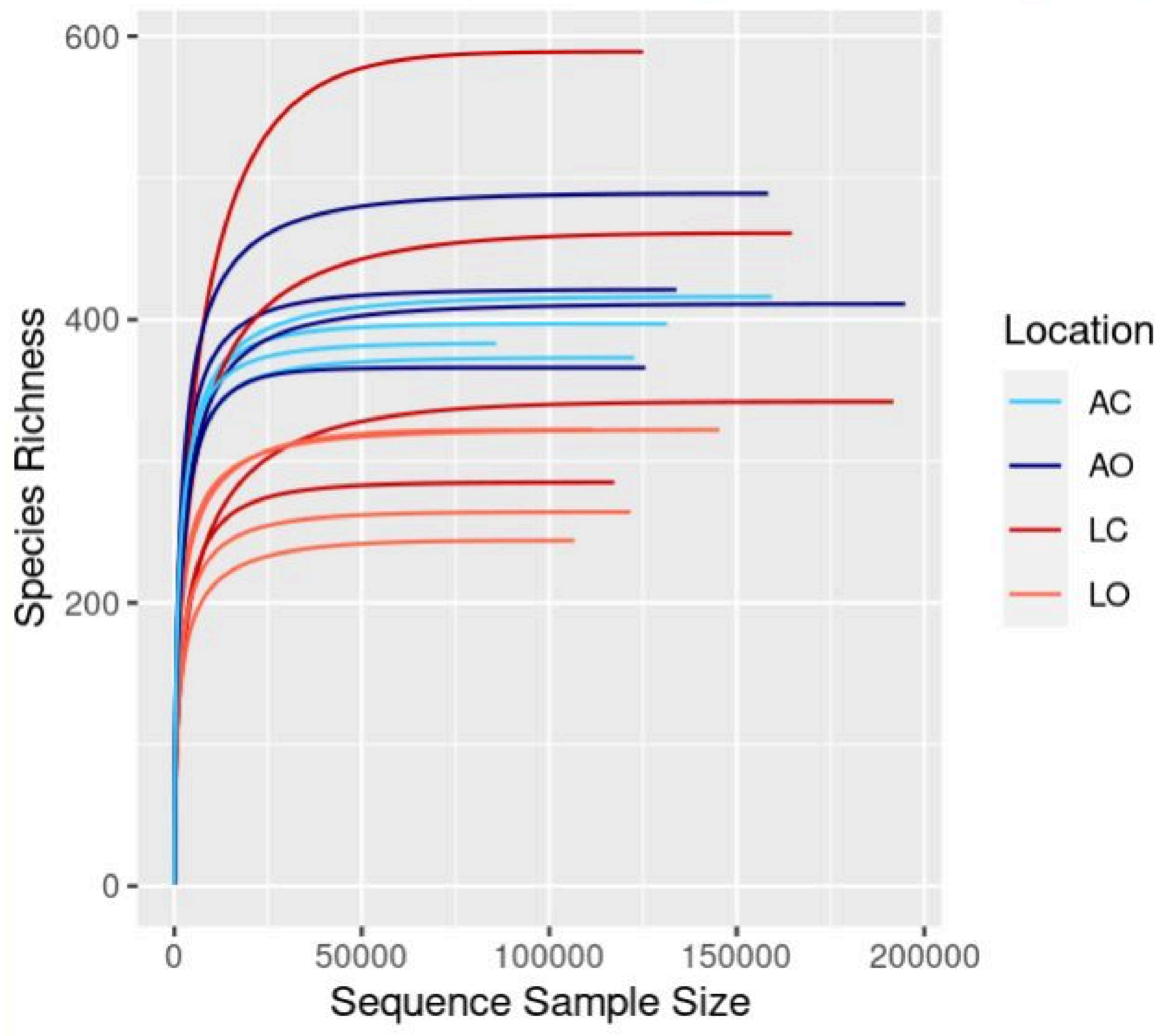


***Fig. S3*** Rarefaction analysis of prokaryotic communities in different locations. Each curve represents a different location (AC, AO, LC, LO). Cold colors represent the Adriatic Sea (light blue coastal site AC; navy blue open site AO) warm colors represent the Ligurian Sea (dark red coastal site LC; light red open site LO)

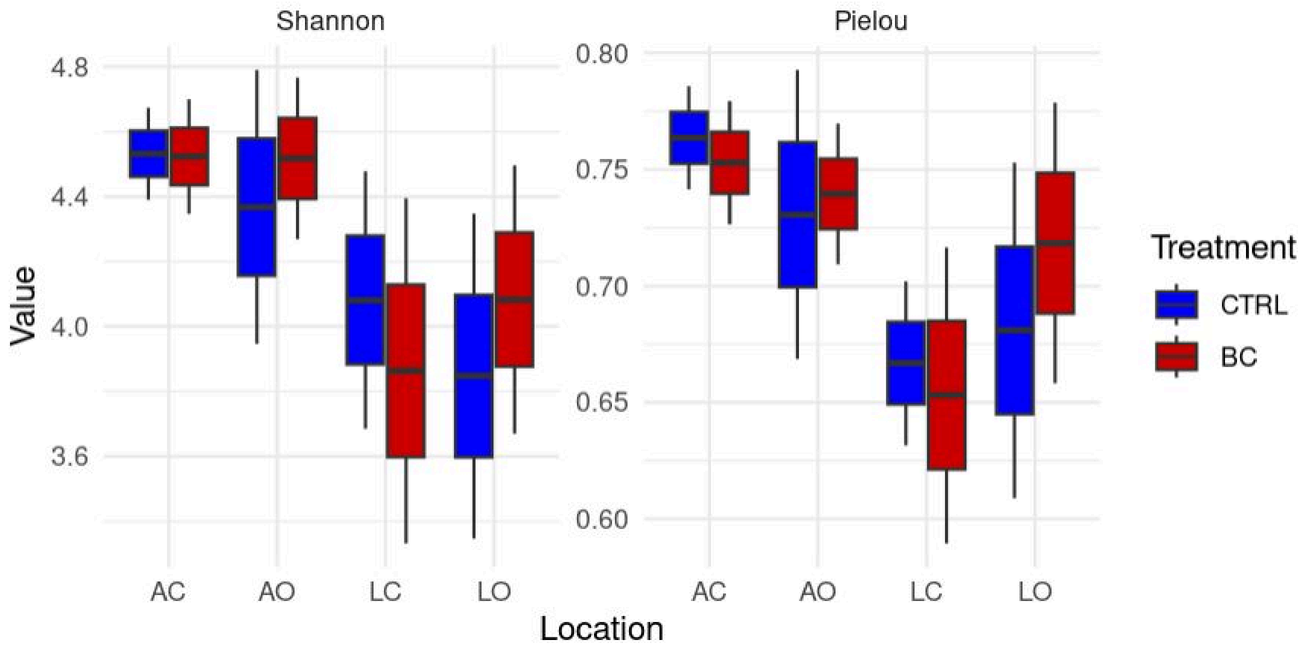


***Fig. S4*** Alpha diversity indices (Shannon index on the left panel and Pielou index on the right panel) of microbial communities from different stations of the Adriatic and Ligurian Sea (AC, AO, LC, LO). Color indicates treatment: black carbon (BC, red) and control (CTRL, blue)

Shannon's diversity index (Fig. S4) showed higher values for the Adriatic Sea (4.49) than for the Ligurian Sea (3.97), thus suggesting the microbial diversity was different in the two areas. For the Shannon index, the ANOVA indicated that the location (Adriatic and Ligurian Sea) showed significance difference ($p = 0.0407 < 0.05$) but neither the treatment ($p = 0.8630$) nor the combined treatment and location factors (p-value $> 0.05$) were significant. Pielou's evenness index (Fig. 6) showed higher values for the Adriatic Sea (0.74) than for the Ligurian Sea (0.68). For Pielou's evenness, a similar pattern was observed. The ANOVA showed that location showed a somewhat significant difference between the areas ($p = 0.0514$) but not the treatment ($p = 0.8621$) nor the combination treatment and location ($p = 0.8399$).

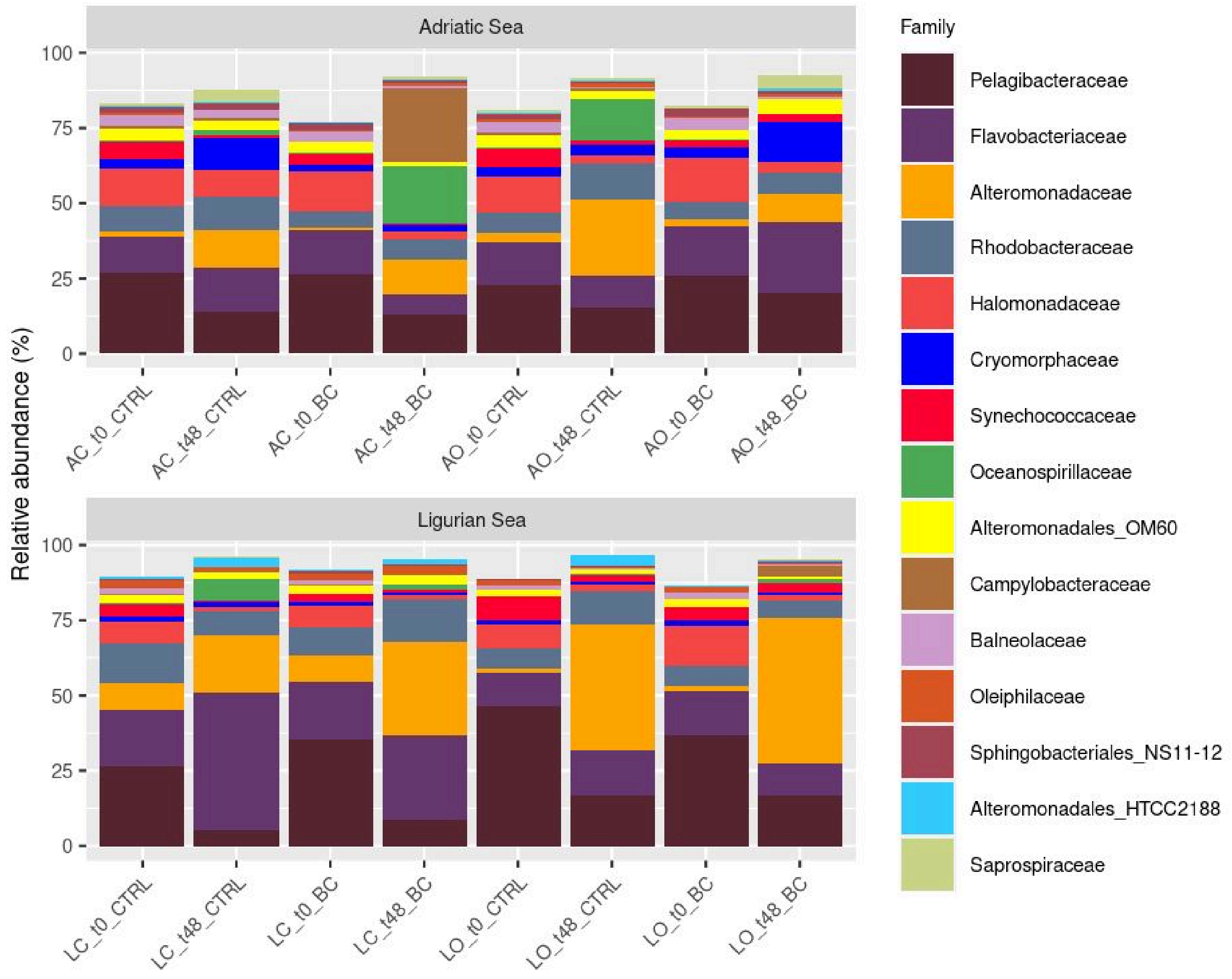


***Fig. S5*** Taxonomic composition, Relative Abundance %, of the most abundant ASVs of the top 15 Family (agglomerated to Family level) in the treatments (BC, CTRL) at different stations (open, coastal) and incubation time (t0, t48) in the Adriatic and Ligurian Sea

**BC PILOT EXPERIMENT:**

**High-resolution imaging by AFM and LSCM and inhibition of motility assay of BC isolated bacterial strains**

On the 29th April 2013, seawater was collected from the Ellen Browning Scripps Memorial Pier (32-52'00" N, 117-15’21" W) with an acid washed bottle. Subsequently, in a flow laminar hood, the water was gravity filtered onto 3 µm in order to remove larger organisms and detrital particles. 500 mL acid washed Nalgene bottles were set as control (CTRL) and BC amended (BC), both in duplicate. BC was spiked at 24 mg $L^{-1}$. The bottles were incubated still at *in situ* temperature 19ºC, under 12:12 light:dark cycle for 48 hours. Illumination was with cool white light at 80 µE $m^{-2}$ $s^{-2}$. Samples were taken to follow the microbial dynamics over time. High resolution imaging at the Atomic Force Microscope (AFM) and Laser Scanning Confocal Microscope (LSCM) was performed as well at T24 from the BC treatment. At the end of the incubation experiments T 48, 100 µL of water from the BC treatment were plated onto ZoBell medium to isolate putative BC resistant microbes. Two bacterial strains were purified (BCR and BCS) and their 16S rRNA gene was sequenced and deposited in NCBI GenBank (BCS: KM112085; BCSR: KM112086). We tested the BC toxicity operationally defined as inhibition of motility on the motile BCR and BCS strains at the dark field microscope at 10X following Grossart et al. 2000. The two strains were exposed to increasing concentration of BC (0, 0.1, 1, 10 mg $L^{-1}$) over 45 minutes. A drop of the bacterial solution was placed in a chambered concave slide and 1 min movies were recorded. Motility was compared against a no BC solution. Briefly, we followed the protocol of Nobel and Furhman (1998) and Patel et al. 2007 using Anodisc filters for SYBR Green I staining and counting at the 1000x epifluorescence microscope using blue light (488 nm excitation, 520 nm emission) the heterotrophic prokaryotes and viruses. *Synechococcus* cells were discriminated from the heterotrophic prokaryotes based on their autofluorescence signals (Malfatti and Azam, 2009). Atomic Force Microscopy imaging was performed on a Dimension FastScan AFM (Bruker, Santa Barbara, CA USA) in PeakForce TappingTM Mode using ScanAsyst-Air probes (nominal spring constant: 0.4 N/m and tip radius: 5 nm, Bruker) for fixed and air-dried samples at the Scripps Institution of Oceanography, UCSD, in the Azam laboratory. The samples were prepared following Malfatti and Azam (2009), in brief 100 µL of formalin fixed sample from BC bottle was spotted onto cleaved mica, then let

it dry and then washed with HPLC water. BC stock solution was also processed in the same way and imaged. 5 scans per sample kind were acquired. Raw AFM height image data were processed using the Nanoscope Analysis built-in package (Bruker), with minimal line-flattening and plane-fitting functions. Anodisc filters, prepared as mentioned above, were imaged at the Laser Scanning Confocal Microscope A1R (Nikon) at 1000X with a 488 nm and 561 nm lasers.

**Results:**

In these experimental settings, viruses decreased upon the BC amendment (Fig. S8), whereas we didn't detect differences in the trends between the BC *vs* control bottles for the heterotrophic prokaryotes and *Synechoccocus* cells. At T48, there were more cultivable bacteria in the BC treatments (Fig.S9).

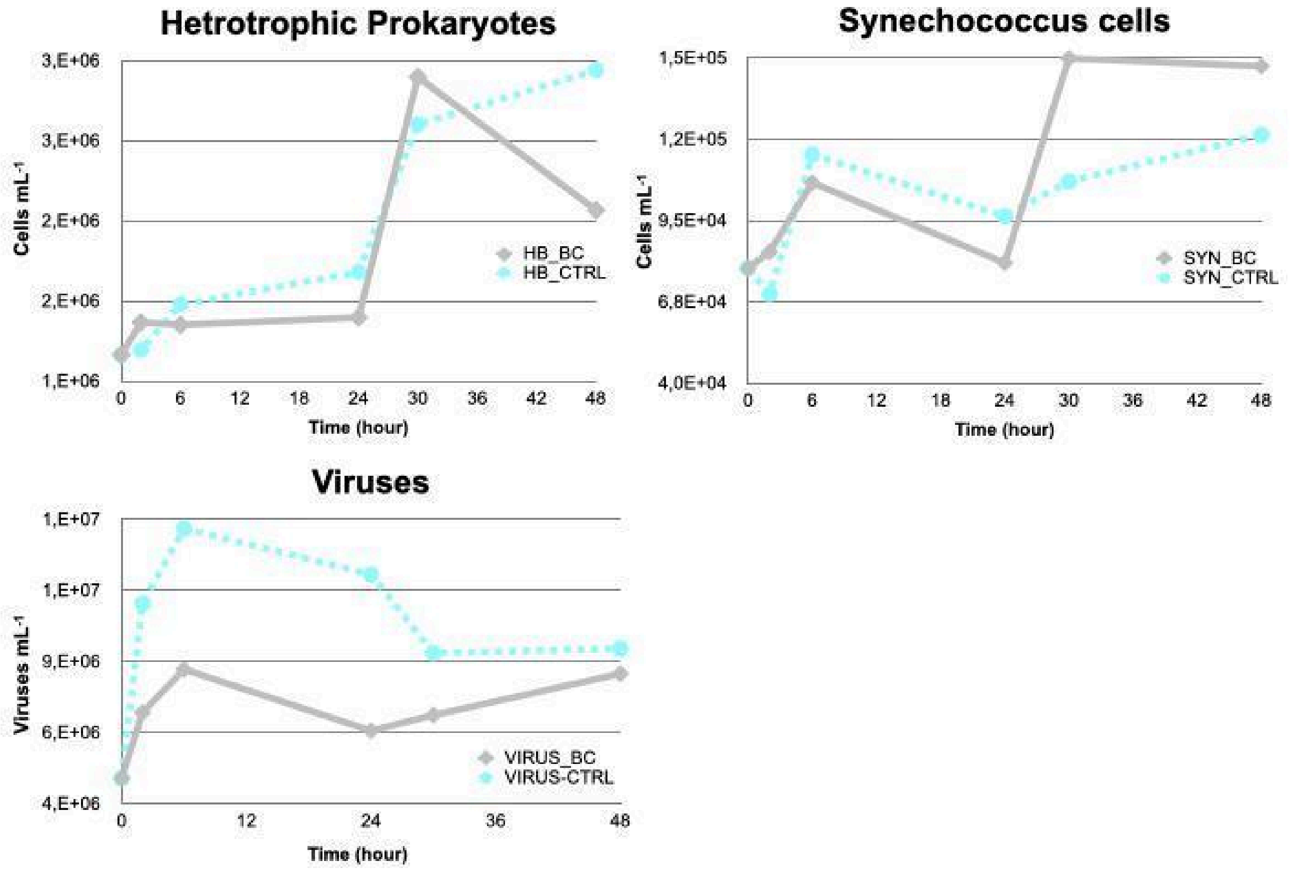


***Fig. S6*** Microbial dynamics, average values, over time in the BC and control (CTRL) duplicates bottles

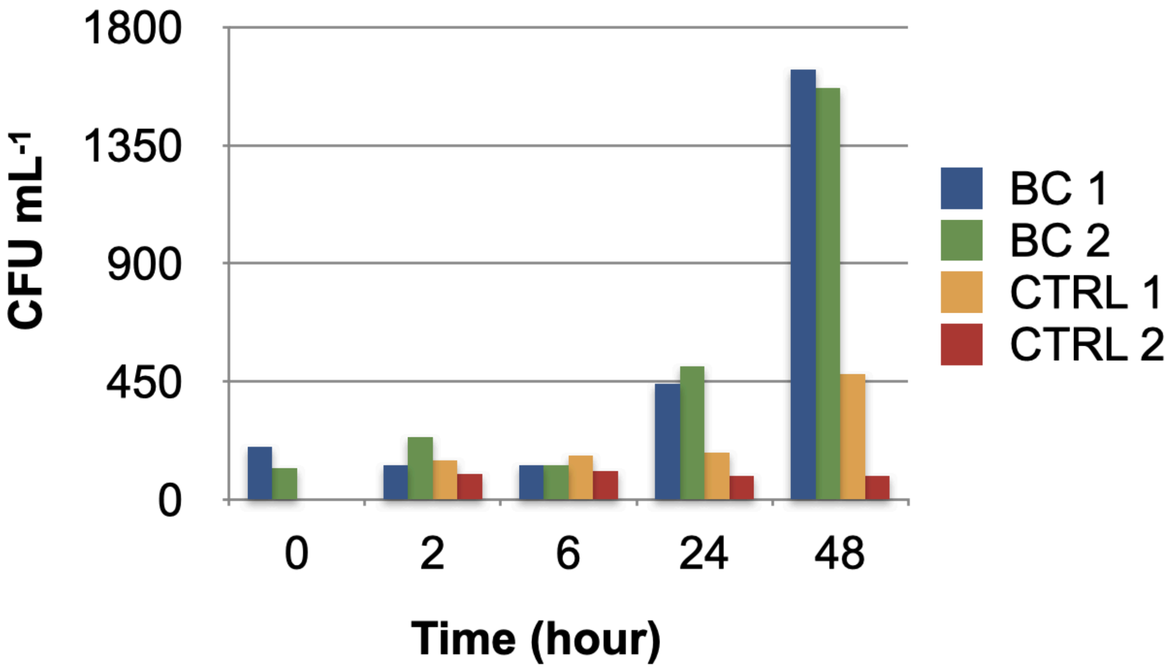


***Fig. S7*** Average Colony Forming Units, CFU $mL^{-1}$ over time in the BC and control (CTRL) duplicates bottles

High-resolution imaging by LSCM showed that viruses and prokaryotes were attached to the BC particles (Fig. S10). BC particles were heterogeneous in shape and size.

The two bacterial strains were isolated from the BC treated samples at T24. By Sanger sequencing of the 16S rRNA gene, they were identified as *Pseudoalteromonas* (BCR) and *Alteromonas* (BCS). When exposed the two isolates to a BC gradient in order to test the effect of BC on motility over 45 min (Table S6). BCS motility was inhibited at the highest concentration of BC (10 mg $L^{-1}$ ), and it remained slightly motile at the intermediate concentrations, whereas BCR motility was not affected.

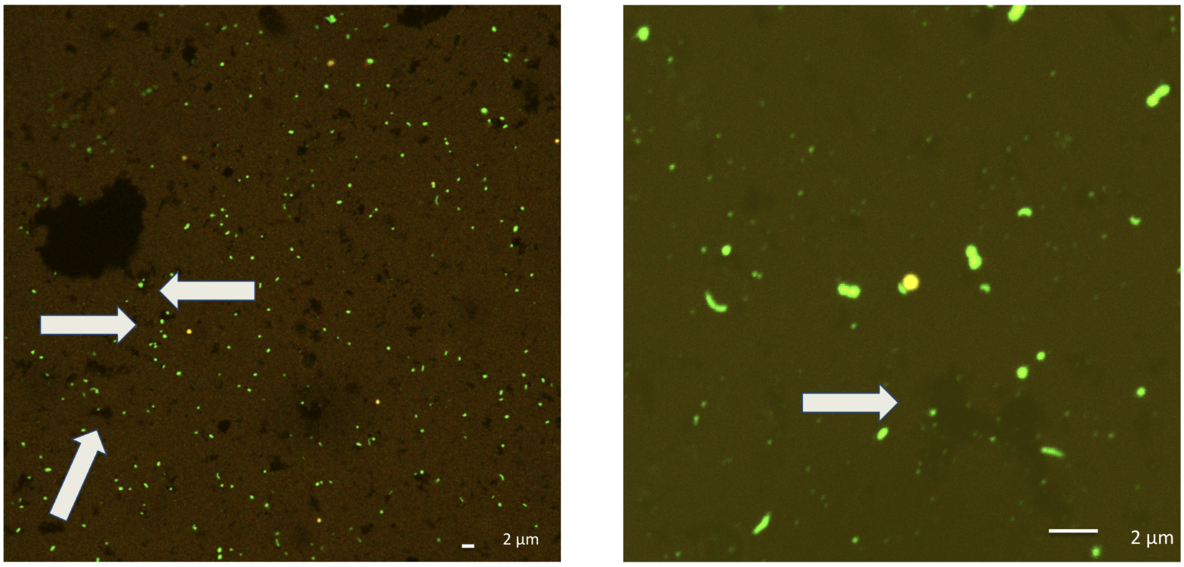


***Fig. S8*** LSCM images of BC samples from T 24. Left panel is 1000x final magnification; right panel is 1000x final magnification with 2x digital magnification

**Table S6** Inhibition of motility assay measured at the dark field microscope for the BCR and BCS bacterial strains along a BC gradient

| Motility | | | | | | |
|---|---|---|---|---|---|---|
| | | Time (min) | | | | |
| | BCR | 0 | 10 | 20 | 45 | |
| Black Carbon | 10 $mgL^{-1}$ | +++ | +++ | +++ | +++ | |
| | 1 mg $L^{-1}$ | +++ | +++ | +++ | +++ | |
| | 0.1 mg $L^{-1}$ | +++ | +++ | +++ | +++ | |
| | Control | +++ | +++ | +++ | +++ | |
| | | | | | | |
| | | Time (min) | | | | |
| | BCS | 0 | 10 | 20 | 30 | 45 |
| Black Carbon | 10 $mgL^{-1}$ | + | - | - | - | - |
| | 1 mg $L^{-1}$ | + | + | + | + | + |
| | 0.1 mg $L^{-1}$ | + | + | + | + | + |
| | Control | +++ | +++ | +++ | +++ | +++ |
